# Functional, Antioxidant, Antibacterial, and Antifungal Activity of Edible Flowers

**DOI:** 10.3390/antiox13111297

**Published:** 2024-10-25

**Authors:** Elena Coyago-Cruz, Alejandro Alarcón, Aida Guachamin, Gabriela Méndez, Edison Osorio, Jorge Heredia-Moya, Johana Zuñiga-Miranda, Elena Beltrán-Sinchiguano, Edwin Vera

**Affiliations:** 1 Carrera de Ingeniería en Biotecnología de los Recursos Naturales, Universidad Politécnica Salesiana, Sede Quito, Campus El Girón, Av. 12 de octubre N2422 y Wilson, Quito 170109, Ecuador; 2 Maestría en Productos Farmacéuticos Naturales, Universidad Politécnica Salesiana, Sede Quito, Campus El Girón, Av. 12 de octubre N2422 y Wilson, Quito 170109, Ecuador; 3 Centro de Investigación Biomédica (CENBIO), Facultad de Ciencias de la Salud Eugenio Espejo, Universidad UTE, Quito 170527, Ecuador; 4 Facultad de Ciencias de la Ingeniería e Industrias, Universidad UTE, Quito 170527, Ecuador; 5 Departamento de Ciencia de los Alimentos y Biotecnología, Facultad de Ingeniería Química, Escuela Politécnica Nacional, Quito 170524, Ecuador

**Keywords:** carotenoids, phenols, organic acid, microextraction, in vitro, PCA

## Abstract

Edible flowers have been used since ancient times, but their potential for improving human health has not been explored. This study aimed to evaluate the profile of bioactive compounds (organic acids, phenolics, and carotenoids) and the antioxidant and antimicrobial activity of nine flower varieties with high concentrations of carotenoids or total phenolic compounds. Ninety-three edible flowers were analysed for physicochemical characteristics, total phenolic and carotenoid concentrations, and antioxidant activity (ABTS). Bioactive profiles were determined by rapid resolution liquid chromatography (RRLC), and antimicrobial activity was determined against *Escherichia coli*, *Staphylococcus aureus*, *Pseudomonas aeruginosa* and *Streptococcus mutans*, and *Candida albicans* and *Candida tropicalis. Chrysanthemum x hybrid* orange, *Helianthus annuus* yellow, *Tagetes patula* orange, *Canna indica* red, and *Hibiscus rosa-sinensis* (orange1 and yellow) showed significant concentrations of total carotenoids. In contrast, *Pelargonium hortorum* orange2, *Hibiscus rosa-sinensis* red1, and *Rosa x hybrid* variety medium yellow showed high levels of total phenolics. The predominant compounds in these species were citric acid (991.4 mg/g DW in *Hibiscus rosa-sinensis* red1), 4-hydroxybenzoic acid (936.2 mg/100 g DW in *P. hortorum* orange2), kaempferol (971. 9 mg/100 g DW in *T. patula* orange), quercetin glucoside (958.8 in *C. x hybrid*), quercetin (919.3 mg/100 g DW in *T. patula*), α-carotene, and β-carotene in *T. patula* orange (989.5 and 601.2 mg/100 g DW, respectively). Regarding antimicrobial activity, *T. patula* orange and *P. hortorum* orange2 inhibited bacterial growth, while *C. x hybrid* orange and *P. hortorum* orange2 inhibited *Candida albicans,* and the latter inhibited *Candida tropicalis*. These results indicate the potential of edible flowers as a natural source of bioactive compounds and as a tool in the fight against antimicrobial resistance.

## 1. Introduction

Interest in natural products has grown significantly in recent decades, driven mainly by the search for safer and more sustainable alternatives to synthetic compounds in the food, cosmetics, and pharmaceutical industries. Thus, throughout history, flowers have been appreciated for their beauty and role in natural medicine and their ability to enrich gastronomy with unique colours, flavours, and textures. Their use has been documented in traditional Middle Eastern, European, and Asian cuisines, and the renewed interest in edible flowers responds to the growing demand for natural, healthy foods rich in bioactive compounds [1,2].

Today, edible flowers are used in culinary applications, from garnishing cocktails to flavouring jellies, wines, vinegars, nutraceutical drinks, and infusion and herbal teas. Their versatility in cooking, together with their potential health benefits, has increased their popularity [3,4].

From a nutritional point of view, edible flowers are a rich source of minerals, vitamins, flavonoids, and other bioactive compounds with antioxidant properties [5]. These substances contribute to the proper functioning of the nervous, cardiovascular, immune, and gastrointestinal systems, and have anti-inflammatory, hepatoprotective, neuroprotective, and anticarcinogenic effects. Antioxidant properties are significant because they neutralise free radicals, which are implicated in developing chronic diseases such as cancer, cardiovascular disease, and premature ageing. However, some flowers also contain antinutrients such as oxalates and phytates, which can interfere with the absorption of essential minerals, so moderate consumption is recommended to avoid nutritional imbalances [5,6,7].

In addition to their antioxidant effects, edible flowers are a promising source of antimicrobial compounds. In a global context where microbial resistance to certain drugs poses a growing threat to public health, bioactive compounds derived from flowers offer natural alternatives to combat bacterial, viral, and fungal infections. Phenolic compounds, essential oils, and other phytochemicals present in these flowers are responsible for their antimicrobial activity [7,8]. For example, *Helichrysum italicum* contains essential oils and polyphenols that are effective against Gram-positive bacteria such as *Staphylococcus aureus*, Gram-negative bacteria such as *Klebsiella pneumoniae*, and yeasts such as *Saccharomyces cerevisiae* [9]. *Pingyin rosebud* extract has shown significant antibacterial activity against *S. aureus* [10]. *Camellia japonica* (var. Carolyn Tuttle) flowers have shown remarkable antimicrobial activity against foodborne pathogens such as *S. aureus*, *Pseudomonas aeruginosa*, and *Salmonella enteritidis*. However, they were ineffective against *Escherichia coli*, *Staphylococcus epidermidis*, and *Bacillus cereus* [11]. In addition, various edible flowers such as roses, marigolds, and chrysanthemums are rich in phenolic compounds such as quercetin and gallic acid, which contribute to their antibacterial, antiviral, and antifungal properties [7].

Despite the promising properties, the efficacy of edible flowers as antioxidants and antimicrobials can vary considerably depending on species, agronomic, environmental, and processing factors [6,12]. Therefore, further research is needed to better understand their bioactive compounds’ mechanisms of action. This study screened 93 edible flower species for carotenoids and total phenolics, and antioxidant activity. Six species with high concentrations of total carotenoids and three with high concentrations of phenolic compounds were selected. In these nine species, a detailed analysis of the profiles of bioactive compounds such as organic acids, phenolic compounds, and carotenoids was carried out to identify the major compounds. In addition, their antibacterial and antifungal activities were evaluated. Thus, this research aimed to contribute to the fight against the resistance of certain micro-organisms by integrating the antioxidant and antimicrobial potential of edible flowers. The results will also provide practical alternatives for the food and pharmaceutical industries, encouraging the development of natural products with benefits for human health. 

## 2. Materials and Methods

### 2.1. Reagents and Standards

The chemicals used in this investigation included acetone (CAS 67-64-1), dichloromethane (CAS 67-66-3), and fluconazole (86386-73-4) reagent grade. At the same time, acetonitrile (CAS 75-05-8), ethanol (CAS 64-17-5), ethyl acetate (CAS 141-78-6), and methanol (CAS 67-56-1) were HPLC-grade and were purchased from Fisher Chemical (Fischer Scientific Inc., Madrid, Spain). In turn, ABTS (2,2-azino-bis-(3-ethylbenzothiazoline-6-sulfonic acid) (CAS 30931-67-0), *DL*-homocysteine (CAS 454-29-5), formic acid (CAS 64-18-6), Folin-Ciocalteu (CAS 7732-18-5), metaphosphoric acid (CAS 37267-86-0), methyl tert-butyl ether (CAS 1634-04-04), potassium hydroxide (CAS 1310-58-3), potassium persulphate (CAS 7727-21-1), sodium carbonate (CAS 497-19-8), sodium hydroxide (CAS 1310-73-2), and sulphuric acid (CAS 7664-93-9), all of analytical grade, were purchased from Sigma (Merck, Darmstadt, Germany). Hydrochloric acid (CAS 7647-01-0) was also obtained in analytical grade from Labscan (RCI Labscan group, Dublin, Ireland). Brain heart infusion (BHI), Mueller–Hinton agar (MHA), and Sabouraud dextrose agar (SDA) were purchased from BD DifcoTM (Fisher Scientific Inc., Madrid, Spain). Yeast peptone cextrose broth (YPDB) was purchased from SRL (Sisco Research Laboratories Pvt. Ltd., Mumbai, India) and streptomycin sulphate (CAS 3810-74-0) was purchased from Phytotech (PhytoTechnology Laboratories^®^, Lenexa, KS, USA). Water was purified using a NANOpureDiamondTM system (Barnsted Inc., Dubuque, IO, USA).

Standards such as citric acid 100.8% (CAS 77-92-9), malic acid 99.0% (CAS 97-67-6), *L*-(+)-tartaric acid 99.5% (CAS 87-69-4), caffeic acid 98.0% (CAS 331-39-5), chlorogenic acid 95.0% (CAS 327-97-9), chrysin 97.0% (CAS 480-40-0), *p*-coumaric acid 98.0% (CAS 501-98-4), *m*-coumaric acid 99.0% (CAS 588-30-7), *o*-coumaric acid 97.0% (CAS 614-60-8), ferulic acid 100.0% (CAS 1135-24-6), gallic acid 100.0% (CAS 149-91-7), *p*-hydroxybenzoic acid 99.0% (CAS 99-06-3), 3-hydroxybenzoic acid 99.0% (CAS 99-06-3), 2,5-dihydroxybenzoic acid 98.0% (CAS 490-79-9), kaempferol 97.0% (CAS 520-18-3), luteolin 98% (CAS 491-70-3), naringin 95.0% (CAS 10236-47-2), quercetin 95.0% (CAS 849061-97-8), rutin 94.0% (CAS 153-18-4), shikimic acid 99.0% (CAS 138-59-0). 0.0% (CAS 138-59-0), syringic acid 95.0% (CAS 530-57-4), vanillic acid 97.0% (CAS 121-34-6), β-carotene 93.0% (CAS 7235-40-7), β-cryptoxanthin 97.0% (CAS 472-70-8), lutein (CAS 127-40-2), lycopene (CAS 502-65-8), zeaxanthin (CAS 144-68-3), and Trolox 98% (CAS 53188-07-1) were purchased from Sigma (Merck, Darmstadt, Germany). *Staphylococcus aureus* ATCC 6538P, *Escherichia coli* ATCC 8739, *Pseudomonas aeruginosa* ATCC 9027, *Streptococcus mutans* ATCC 25175, *Candida albicans* ATCC 1031, and *Candida tropicalis* ATCC 13803 were purchased from ATTC (ATTC, Manassas, VA, USA).

### 2.2. Physicochemical Quantification

This study considered ninety-three edible flowers grown in different regions of Ecuador (Table 1). For the physicochemical characterisation, thirty fresh flowers of each species were collected and analysed for weight, size, pH, soluble solids, total titratable acid, moisture, and ash. Petals from about one hundred flowers were stored and frozen at −21 °C and then freeze-dried in a Christ Alpha 1-4 LDplus (Martin Gefriertrocknungsanlagen GmbH, Osterode am Harz, Germany). The dried petals were ground to a fine powder and stored in amber glass jars under a nitrogen atmosphere until analysis.

Colour was measured on fresh flowers with a CR-400 tristimulus colour meter (Konica Minolta Sensing Americas, Ramsey, NJ, USA) using the CIELAB scale (L*, a* and b*) [13]. Flowers were weighed using an ML204T/00 balance (Mettler Toledo, Columbia, MD, USA) and equatorial and longitudinal diameters were measured with a digital calliper. pH measurements were made with a SevenMulti TM electronic pH meter (Mettler Toledo, Columbia, MD, USA) according to the ISO-1842 method [14]. Soluble solids were quantified by a Hitech portable refractometer (Hi-tech RHB-32ATC, Río de Janeiro, Brasil) according to US-ISO-2173 [15]. Total titratable acidity was determined according to US-ISO-750:1998 [16]. Moisture and ash content were quantified by gravimetric methods using a Memmert Be 20 oven (Memmert GmbH+Co.KG, Schwabach, Germany) at 110 °C and a Thermolyne muffle (Thermo Fisher Scientific, Waltham, MA, USA) at 525 °C, respectively [13,17].

### 2.3. Quantification of Total Carotenoids

Microextraction was performed in the dark and in triplicate. A total of 20 mg of lyophilised powder was mixed with a 300 uL mixture of acetone, methanol, and dichloromethane (1:1:2). This mixture was homogenised in a VM-300 vortexer (Interbiolab Inc., Orlando, FL, USA) and vortexed for one minute in a Fisher Scientific FS60 ultrasonic bath (Fisher Scientific, Waltham, MA, USA). The mixture was centrifuged at 14,000 rpm for 3 min at 4 °C in a MiniSpin microcentrifuge (Eppendorf, Bochum, Germany). The organic phase was collected, and the extraction process was repeated until the solid residue became colourless. The coloured phase was evaporated to dryness on a Buchi TM R-100 rotary evaporator (Fisher Scientific, Waltham, MA, USA) at below 30 °C.

The dried extract was dissolved in 2 mL of HPLC-grade ethanol to quantify total carotenoids. This was transferred to a 10 mm light path quartz cell, and the absorbance was measured at 450 nm using a ThermoSpectromic Genesys 10 UV-Vis spectrophotometer (ThermoFisher Scientific, Waltham, MA, USA). The concentration was assessed using a calibration curve with 5 mg β-carotene dissolved in 25 mL ethanol. The concentration of total carotenoids in the samples was expressed as micrograms of β-carotene per 100 g dry weight (DW) (µg β-carotene/100 g DW) [18].

### 2.4. Quantification of Total Phenolic Compounds

Microextraction was performed in triplicate. A total of 40 mg of lyophilised powder was mixed with 1 mL of 80% methanol acidified with 0.1% hydrochloric acid. The mixture was homogenised in a VM-300 vortexer (Interbiolab Inc., Orlando, FL, USA) and vortexed for 2 min in a Fisher Scientific FS60 ultrasonic bath (Fisher Scientific, Waltham, MA, USA). The supernatant was separated by centrifugation at 14,000 rpm for 5 min at 4 °C in a MiniSpin microcentrifuge (Eppendorf, Bochum, Germany). This extraction process was repeated twice, using 500 µL of acidified methanolic solution in each step. The collected supernatant was filtered through a 0.45 µm PVDF filter, and the resulting solution was kept frozen until analysis [12,19].

To quantify total phenolic compounds, 20 µL of the filtered supernatant was added to a 96-well VWR tissue culture plate (Novachen, Pittsburgh, PA, USA) with 100 µL of a 1:4 Folin–Ciocalteu solution and homogenised. After 4 min, 75 µL of a sodium carbonate solution (100 g/L) was added and shaken for 1 min. The mixture was then allowed to stand for two hours at room temperature, and the absorbance was measured at 750 nm using a BioTek Synergy H1 microplate reader (Agilent Scientific Instruments, Santa Clara, CA, USA). A calibration curve was established with gallic acid in a concentration range between 10 and 200 mg/L. The concentration of total phenolics was expressed as mg gallic acid equivalent per 100 g dry weight (mg GAE/100 g DW) [20].

### 2.5. Antioxidant Activity

For extraction, 20 mg of lyophilised powder was mixed with 400 µL of methanol and 400 µL of distilled water. The mixture was homogenised by vortexing and shaken in an ultrasonic bath for 3 min. The supernatant was separated by microcentrifugation at 14,000 rpm for 5 min at 4 °C. The resulting solid was mixed with 560 µL of acetone and 240 µL of distilled water. The process was repeated to obtain the supernatant, which was combined with the previous supernatant, and this final mixture was refrigerated until analysis.

For the quantification of antioxidant activity, the ABTS•+ radical was prepared by mixing a 1:1 solution of 7 mM ABTS with 2.45 mM potassium persulfate and allowed to stand in the dark for 16 h. The radical was diluted 1/10 with absolute ethanol or until an absorbance of 0.7 at 0.4 nm was obtained [13]. A 2.5 nM Trolox stock solution was prepared for the calibration curve and diluted to 75, 50, 25, and 12.5% concentrations. Again, 20 µL of the final extract was added to a 96-well plate containing 280 µL of ABTS•+ radical solution. Absorbance was measured at 754 nm using a spectrophotometer with a Thermo Scientific Multiskan GO microplate reader (Agilent Scientific Instruments, Santa Clara, CA, USA), and antioxidant activity was expressed as mmol Trolox equivalents per gram dry weight (mmol TE/100 g DW) [17].

### 2.6. Bioactive Compound Profiles

Six samples with the highest concentrations of total carotenoids and three with the highest concentrations of total phenolics were extracted using the following methods.

#### 2.6.1. Organic Acid Profile

Extraction was performed in triplicate. A total of 40 mg of lyophilised powder was mixed with 1.5 mL of 0.02 N sulphuric acid containing 0.05% metaphosphoric acid and 0.02% *DL*-homocysteine. The mixture was homogenised by vortexing, shaken in an ultrasonic bath for 3 min, and made up to 2 mL with deionised water. The supernatant was separated by centrifugation at 14,000 rpm at 4 °C for 5 min and filtered on a 0.45 µm PVDF filter. The filtered extract was placed in a vial for injection into an RRLC 1200 liquid chromatograph equipped with a DAD-UV-VIS detector at a wavelength of 210 nm and a YMC-Triart C18 column (150 × 4.6 mm, 3 µm, 12 nm, 400 bar) (YMC Europe GmbH, Dinslaken, Germany). The column temperature was maintained at 30 °C, and the flow rate was 1 mL/min under isocratic conditions. The mobile phase was a 0.027% sulphuric acid solution, and the run time was 30 min with an injection volume of 20 µL. Individual identification of the organic acids was performed by comparison of retention times, UV-Vis spectra, and an internal standard. Chromatograms were monitored at 210 nm using the Open Lab ChemStation software (version 2.15.26). Quantification of organic acids was performed using external calibration curves containing a concentration of 100 mg/mL of citric, malic, and *L*-(*+*)-tartaric acid standards, prepared and quantified separately with injection volumes of 3, 5, 10, 15, and 20 µL [13]. Organic acid content was expressed as milligrams per gram dry weight (mg/g DW).

#### 2.6.2. Phenol Profile

Six samples with the highest concentrations of total carotenoids and three with the highest concentrations of total phenolics were re-extracted using the method described in Section 2.4. For the quantification of phenolic profiles, 20 µL of the filtered methanolic extract was placed in a vial for injection into an Agilent 1200 series RRLC liquid chromatograph coupled to a DAD-UV-Vis detector with a wavelength scan between 220 and 500 nm [8]. The phenolic compounds were separated using a Zorbax Eclipse Plus C18 column (4.6 × 150 mm, 5 µm) (Agilent Technologies, Santa Clara, CA, USA) at 30 °C. The mobile phase consisted of a 1 mL/min flow of a 0.01% aqueous solution of formic acid (solvent A) and acetonitrile (solvent B) using a linear gradient of 100% at 0 min; 95% A + 5% B at 5 min; 50% A + 50% B at 20 min; and washing and re-equilibration of the column at 30 min. Phenols were identified using the Open Lab ChemStation software (version 2.15.26) with spectra at 280, 320, and 370 nm as appropriate. For quantification, a calibration curve was constructed using different injection volumes (3, 5, 10, 15, and 20 µL) of a 1 mg/mL solution of caffeic acid, chlorogenic acid, chrysin, *p*-coumaric acid, *m*-coumaric acid, *o*-coumaric acid, ferulic acid, gallic acid, *p*-hydroxybenzoic acid (4-hydroxybenzoic acid), 3-hydroxybenzoic acid, 2,5-dihydroxybenzoic acid, kaempferol, luteolin, naringin, quercetin, rutin, shikimic acid, syringic acid, quercetin glycoside, and vanillic acid. Each phenolic compound was expressed as milligrams per hundred grams of dry weight (mg/100 g DW) [12].

#### 2.6.3. Carotenoid Profile

Six samples with the highest concentrations of total carotenoids and three with the highest concentrations of total phenolics were re-extracted, as described in Section 2.3. The dried extracts were saponified with 500 μL of 30% (*w*/*v*) methanolic potassium hydroxide solution stirred for one hour under N_2_ at 25 °C in the dark. After this time, 500 μL of dichloromethane and 800 μL of a 5% NaCl solution were added, and the mixture was vortex-homogenised and centrifuged at 14,000× *g* for 3 min to remove the aqueous phase with a Pasteur pipette. To remove the hydroxide residue, successive washes with water were performed until the aqueous phase reached a pH of 7. The resulting coloured phase was dried below 30 °C using a rotary evaporator and stored under a nitrogen atmosphere at −20 °C until further analysis.

The dried saponified residue was dissolved in 20 μL ethyl acetate and centrifuged at 13,171× *g*, 4 °C for 3 min. The supernatant was transferred to a vial insert and injected in duplicate into an RRLC 1200 system equipped with a DAD-UV-Vis detector. Analysis was performed on a C30 YMC column (3 µm, 4.6 cm × 150 mm) (Agilent Scientific Instruments, Santa Clara, CA, USA) according to the method described by Stinco et al. [21]. The column was maintained at 30 °C, the flow rate was 1 mL/min, and the injection volumes ranged from 0.5 to 5 µL. The mobile phase consisted of methanol (solvent A), methyl tert-butyl ether (solvent B), and water (solvent C) with a linear gradient elution as follows: 95% A + 5% B + 0% C, 0 min; 95% A + 5% B + 0% C, 5 min; 95% A + 5% B + 0% C, 5 min; 89% A + 11% B + 10% C, 10 min; 89% A + 11% B + 0% C, 10 min; 75% A + 25% B + 0% C, 16 min; 40% A + 60% B + 0% C, 20 min; 15% A + 85% B + 0% C, 22 5 min; 90% A + 5% B + 5% C, 25 min; and 90% A + 5% B + 5% C, 28 min. The Open Lab ChemStation software (version 2.15.26) processed the chromatograms. Comparison of retention times and UV-Vis spectra identified carotenoids. The chromatograms were analysed at 285, 350, and 450 nm. Quantification of carotenoids was performed using external calibration curves with a concentration of 1 mg/mL standard of β-carotene, β-cryptoxanthin, lutein, lycopene, and zeaxanthin. These standards were prepared and quantified separately with 3, 5, 10, 15, and 20 µL injection volumes. Carotenoid concentrations were expressed as milligrams per 100 g dry weight (mg/100 g DW).

### 2.7. Antimicrobial Activity

#### 2.7.1. Preparation of Flower Extracts

To prepare the extract, 0.2 g of lyophilised sample of the six species with the highest concentrations of total carotenoids and three with the highest concentrations of total phenolics were weighed. A total of 1 mL of 50% ethanol was added to the samples, followed by homogenisation and shaking in an FS60 ultrasonic bath (Scientific, Waltham, MA, USA) for 6 min. The supernatant was separated by centrifugation at 14,000 rpm for 3 min in a microcentrifuge (Eppendorf, Bochum, Germany). The extraction process was repeated twice, using 0.5 mL of the ethanol solution in each repetition. The final supernatant was then filtered through PDVF filters of 0.45 µm and 25 mm diameter. The extract was dried using a Christ Alpha 1-4 LDplus freeze dryer (GmbH, Bochum, Germany). Finally, the dried extract was resuspended in 1 mL of sterile distilled water (Table 2) to determine antimicrobial activity using the well diffusion method according to the Clinical and Laboratory Standards Institute (CLSI) guidelines with some modifications [22,23,24].

#### 2.7.2. Preparation of Inoculum

The antibacterial properties of flowers extracts were tested against Gram-positive bacteria *Staphylococcus aureus* ATCC 6538P, Gram-negative bacteria *Escherichia coli* ATCC 8739, *Pseudomonas aeruginosa* ATCC 9027, *Streptococcus mutans* ATCC 25175, and two pathogenic fungus *Candida albicans* ATCC 1031 and *Candida tropicalis* ATCC 13803. All bacterial strains used in this study were obtained from the American Type Culture Collection (ATCC, Manassas, VA, USA) and were maintained at −80 °C with 25% (*v*/*v*) glycerol supplementation.

The Gram-positive and Gram-negative bacteria were pre-cultured in brain heart infusion (BHI) overnight in a rotary shaker at 37 °C. Afterward, each strain was adjusted at a concentration of 0.5 MacFarland standard (108 cells/mL). The fungal inoculum was prepared from the 24 h old culture of fungal isolates in Yeast Peptone Dextrose Broth (YPDB). Each strain was adjusted to 0.5 MacFarland standard (final concentration of 106 cells/mL).

#### 2.7.3. Well Diffusion Assay

The agar well diffusion method was used to evaluated the antibacterial and antifungal activities of different floral extracts. The suspensions of active micro-organisms were spread uniformly on solidified Mueller–Hinton agar (MHA) for bacteria strains, and over Sabouraud dextrose agar (SDA) for fungal strains, using a sterile swab. Then, agar wells (5 mm diameter) were made on each plate using a sterile cork borer. A fixed volume of about 80 μL with different concentrations of the floral extracts (Table 2) was added to the wells, and Petri plates were incubated at 37 °C/18 h for bacteria and at 35 °C/48 h for fungus. The inhibition zones obtained were measured in millimetres. Streptomycin (1560 µg/mL) and Fluconazol (1250 µg/mL) were used as controls for growth inhibition at a recommended working concentration for bacterial and fungal strains, respectively. Additionally, distilled water was used as a negative control. These assays were performed at least in triplicate.

### 2.8. Statistical Analysis

Statistical analysis was conducted using Statgraphics Centurion XVII, Rstudio 4.3.3, and the Sigmaplot 14.0 software. Results are given as the mean ± standard deviation. A simple ANOVA was employed to identify significant differences, with a significance level set at *p* < 0.05. Furthermore, correlation and principal component analyses explored potential relationships among the study parameters—this analysis aimed to uncover any associations between the variables under investigation.

## 3. Results

### 3.1. Physicochemical Quantification

**Figure 1:**
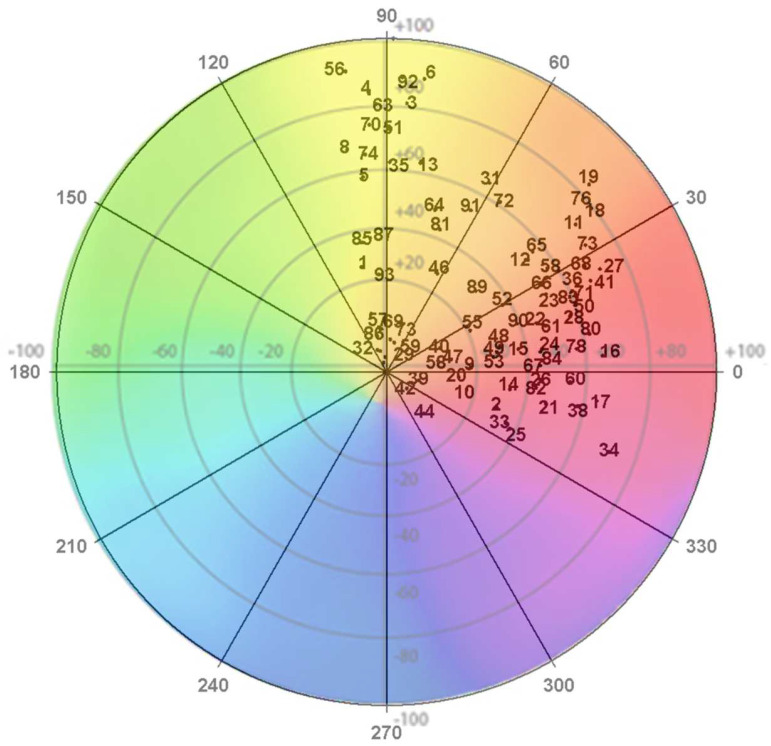
CIELAB colour coordinates of the flowers under study. **Note:** The numbers correspond to the number of blossoms examined (Table 1).

Figure 1 shows the colour distribution in the polar coordinates of the flowers under study. The flowers were primarily red and orange and located in the first, second, and fourth quadrants.

Table S1 shows the results of the physicochemical analyses on the petals studied. This study evaluated weight, size, pH, soluble solids, titratable acidity, moisture, and ash.

Flower weight showed a remarkable variability, ranging from light species such as *Anethum graveolens*, *Lantana camara* multicolor, and *Lantana viburnoides* (red, red-orange, and yellow), with a minimum weight of 0.01 g, to heavier flowers such as *Rosa x hybrid* big red, which reached 23.39 g. Flower size also varied significantly, with the longitudinal diameter ranging from 0.2 cm in *Anethum graveolens* to 12.15 cm in *Hibiscus rosa*-*sinensis* (pink). In comparison, the equatorial diameter ranged from 0.16 cm in *Anethum graveolens* to 15.57 cm in *Dahlia pinnata* orange.

The pH of the flowers analysed varied widely, from 0.8 in *Pelargonium hortorum* pink-(white2) to 13.0 in species such as *Antirrhinum majus* (red), *Dianthus chinensis* (red), *Pelargonium hortorum* (red), and *Raphanus raphanistrum*. In addition, soluble solids ranged from 0.1 °Brix in *Lantana camara* multicolor to 11 °Brix in species such as *Raphanus raphanistrum* and *Rosa x hybrid* roseta big red. The total titratable acidity ranged from 0.06% in *Antirrhinum majus* yellow to 4.69% in *Dianthus chinensis* red. Moisture content showed marked differences between species, ranging from 51.87% in *Pelargonium hortorum* (red-white) to 97.25% in *Begonia doblet* and *Canna indica* (red). As for ash content, this parameter ranged from 0.09% in *Lantana viburnoides* yellow to 9.17% in *Chamaemelun nobile*.

### 3.2. Quantification of Total Carotenoids and Phenols and Antioxidants Activity

Table 3 shows the average concentration of total carotenoids, total phenolics, and antioxidant activity quantified by the ABTS method.

The total carotenoid concentration in this study ranged from 32.38 mg/100 g DW in *Pelargonium hortorum* to 5745.28 mg/100 g DW in *Calendula officinalis*. In addition, species such as *Helianthus annuus* (5154.63 mg/100 g DW), *Lantana camara* multicolor (2947.7 mg/100 g DW), *Hibiscus rosa*-*sinensis* yellow (2468.5 mg/100 g DW), and *Tagetes patula* orange (2057.7 mg/100 g DW) were noted for having total carotenoid concentrations above 2000 mg/100 g DW.

Similarly, the concentration of total phenolics in this study ranged from 16.49 mg GAE/g DW in *Chamaemelun mobile* to 586.37 mg GAE/g DW in *Rosa x hybrid* mini red. Thus, species such as *Rosa x hybrid* mini red (586.3 mg GAE/g DW), *Pelargonium hortorum* orange2 (525.4 mg GAE/g DW), *Rosa x hybrid* medium yellow (363.9 mg GAE/g DW), *Rosa x hybrid* medium pink (361.3 mg GAE/g DW), and *Rosa x hybrid* medium white (359.1 mg GAE/g DW) showed high concentrations of total phenols.

In antioxidant activity, the percentage inhibition showed a significant range, from 4.57% in *Chamaemelun mobile* to 100% in *Citrus x aurantifolia*. Total antioxidant activity also showed significant variations, ranging from 6.01 mmol ET/100 g DW in *Chamaemelun nobile* to 874.81 mmol ET/100 g DW in *Citrus x aurantifolia*. The species with higher concentrations of antioxidant activity included *Ruda chalepensis* (784.56 mmol ET/100 g DW), *Pelargonium hortorum* red-white (785.63 mmol ET/100 g DW), *Pelargonium hortorum* pink (785.79 mmol ET/100 g DW), *Dahlia pinnata* orange (796.35 mmol ET/100 g DW), and *Citrus x aurantifolia* (874.81 mmol ET/100 g DW).

**Figure 2:**
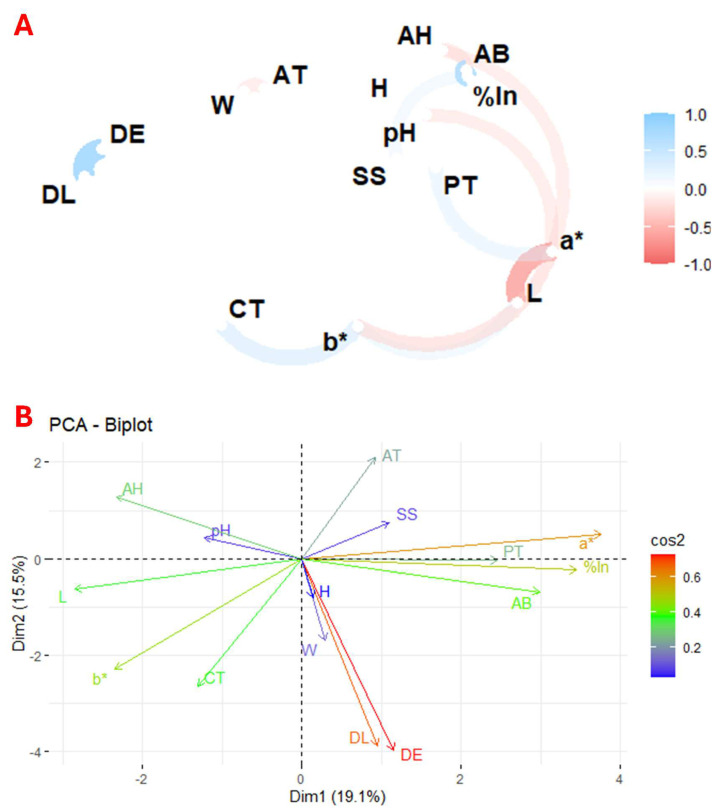
Exploratory multivariate analysis using correlation (**A**) and principal component (**B**) analysis of the 93 flowers under study. Notes: W, weight; DL, longitudinal diameter; DE, equatorial diameter; SS, soluble solids; AT, titratable acidity; H, humidity; AH, ash; a*, colour coordinate; b*, colour; L, colour intensity; CT, total carotenoids; PT, total phenolics; %In, % inhibition; AB, antioxidant activity.

Plant species contain a wide variety of physicochemical characteristics and bioactive compounds, making it difficult to perform a direct comparative statistical analysis between them, even within the same genus. In this context, correlation and principal component analysis are valuable tools to identify the relationships between the different variables and to determine which factors have a more significant impact on the study results. Thus, Figure 2 presents the correlation and principal component analysis (PCA) of the studied variables across the 93 flower species. Figure 2A shows the correlation between the physicochemical parameters, total carotenoids, total phenolics, and antioxidant activity of the flower, highlighting the relationship between these variables. In contrast, Figure 2B depicts the principal components derived from the PCA, which visually represents the distribution and grouping of the flowers based on the variance explained by the key variables.

### 3.3. Bioactive Compound Profiles

Flowers are an important source of bioactive compounds. Thus, Table 4 shows the average concentrations of organic acids (tartaric, malic, and citric acids), phenolic compounds, and carotenoids of the flower species with high concentrations of carotenoids or phenolics.

In the selected flower species, the concentration of organic acids as the sum of individual compounds varied significantly from 50.8 mg/g DW in *Tagetes patula* (7) to 1033.4 mg/g DW in (49). In this context, tartaric acid varied from 11.2 mg/g DW in *Hibiscus rosa*-*sinensis* (51) to 113.8 mg/g DW in *Canna indica* (12). Malic acid ranged from 6.2 mg/g DW in *Hibiscus rosa*-*sinensis* (45) to 119.7 mg/g DW in *Hibiscus rosa*-*sinensis* (49). In comparison, citric acid ranged from 8.9 mg/g DW in *Helianthus annuus* (6) to 991.4 mg/g DW in *Hibiscus rosa*-*sinensis* (49).

For total phenolic compounds, as the sum of individual compounds, concentrations ranged from 175.0 mg/100 g DW in *Helianthus annuus* to 2827.4 mg/100 g DW in *Tagetes patula* (7). High concentrations of individual phenolic compounds have been identified, such as gallic acid with 17.2 mg/100 g DW in *Pelargonium hortorum* (19) and vanillic acid with 0.6 mg/100 g DW in *Helianthus annuus* (6). In addition, 38.9 mg/100 g DW of *p*-coumaric acid was found in *Pelargonium hortorum* (19), 52.0 mg/100 g DW of *m*-coumaric acid in *Hibiscus rosa*-*sinensis* (51), and 3.9 mg/100 g DW of syringic acid in the same species. Other compounds identified include chlorogenic acid (1.2 mg/100 g DW), caffeic acid (3.6 mg/100 g DW), and naringin (120.1 mg/100 g DW) in *Hibiscus rosa*-*sinensis* (51). Also, 13.0 mg/100 g DW of ferulic acid in *Helianthus annuus* (6), 936.2 mg/100 g DW of 4-hydroxybenzoic acid in *Pelargonium hortorum* (19), 854.3 mg/100 g DW of rutin in *Hibiscus rosa*-*sinensis* (51), 971. 9 mg/100 g DW of kaempferol in *Tagetes patula* (7), 958.8 mg/100 g DW of quercetin glycoside in *Chrysanthemum x hybrid* (3), and 919.3 mg/100 g DW of quercetin in *Tagetes patula* (7).

On the other hand, carotenoids as the sum of individual compounds ranged from 2.5 mg/100 g DW in *Hibiscus rosa*-*sinensis* (49) to 2043.3 mg/100 g DW in *Tagetes patula* (7). Among the most prominent individual carotenoids, a concentration of 989.5 mg/100 g DW of α-carotene, 601.2 mg/100 g DW of β-carotene, 34.2 mg/100 g DW of β-cryptoxanthin, 149.5 mg/100 g DW of zeaxanthin, and 10.0 mg/100 g DW of zeinoxanthinwas found in *Tagetes patula*. In addition, 59.5 mg/100 g DW of violaxanthin in *Helianthus annuus* and 300.9 mg/100 g DW of lutein in *Helianthus annuus* were recorded.

### 3.4. Antimicrobial Activity

**Figure 3:**
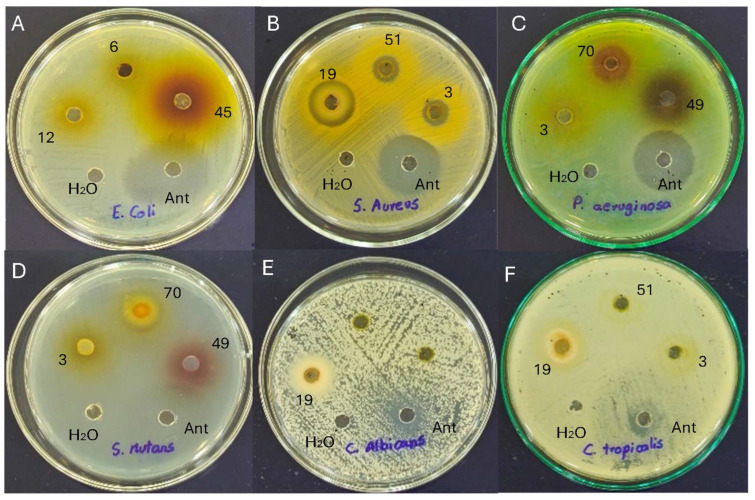
Antimicrobial activity of flower extracts against (**A**) *Escherichia coli*; (**B**) *Staphylococcus aureus*; (**C**) *Pseudomonas aeruginosa*; (**D**) *Streptococcus mutans*; (**E**) *Candida albicans*; and (**F**) *Candida tropicalis*. Note: 3, *C. x hybrid* (orange); 6, *H. annuus* (yellow); 12, *C. indica* (red); 19, *P. hortorum* (orange2); 45, *H. rosa-sinensis* (orange1); 49, *H. rosa-sinensis* (red1); 51, *H. rosa-sinensis* (yellow); 70, *Rosa x hybrid* medium red.

Antimicrobial susceptibility testing is critical for the effective management of pathogenic micro-organisms. The well diffusion method demonstrated the magnitude of the susceptibility of the pathogenic micro-organisms (Figure 3). The mean diameter of the inhibition index containing flower extracts at different concentrations is presented in Table 5.

**Figure 4:**
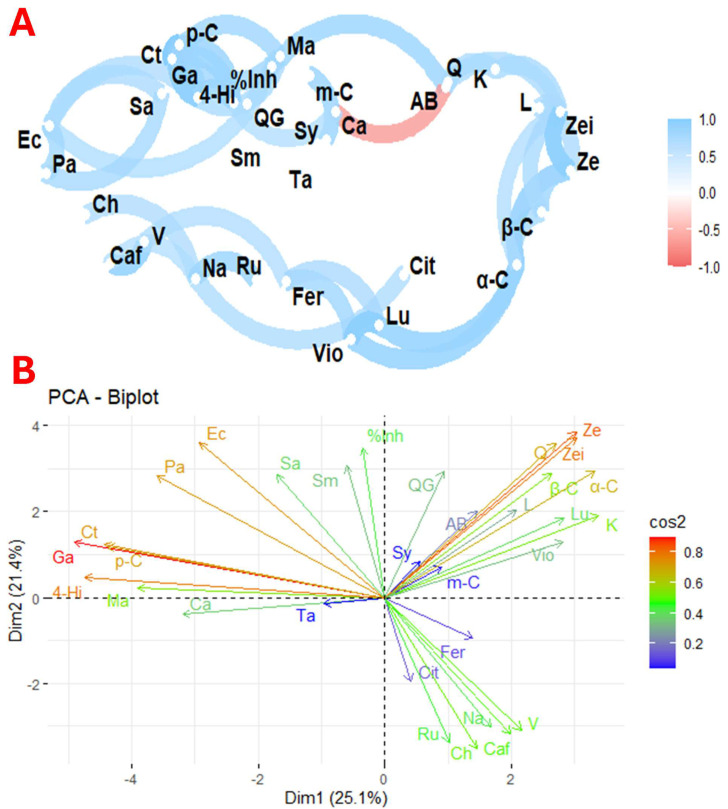
Exploratory multivariate analysis using correlation (**A**) and principal component (**B**) analysis of the nine selected flowers. Notes: %Inh, % inhibition; AB, antioxidant activity; Ta, tartaric acid; Ma, malic acid; Cit, citric acid; α-C, α-carotene; β-C, β-carotene; L, β-cryptoxanthin; L, lutein; Vio, violaxanthin; Ze, zeaxanthin; Zei, zeinoxanthin; Caf, caffeic acid; Ch, chlorogenic acid; Fer, ferulic acid; Ga, gallic acid; 4-Hi, 4-hydroxy benzoic acid; K, kaempferol; L, luteolin; *p*-C, *p*-C; *m*-Cumaric, *m*-cumaric acid; Na, naringin; QG, quercetin glycoside; Q, quercetin; Ru, rutin; Sy, syringic acid; V, vanillic acid; Ec, *Escherichia coli*; Sa, *Staphylococcus aureus*; Pa, *Pseudomonas aeruginosa*; Sm, *Streptococcus mutans*; Ca, *Candiad albicans*; Ct, *Candiad tropicalis*.

Figure 4 illustrates the correlation and principal component analysis of the variables studied across the nine flower species. Figure 4A shows the correlation between the profiles of organic acids, phenolics, carotenoids, and antimicrobial activity of the nine selected species. In contrast, Figure 4B shows the principal components of these selected species.

## 4. Discussion

### 4.1. Physicochemical Quantification

This study observed a large variability in the weight and size of the analysed flowers, even within the same family. For example, in the family Asteraceae, flower weights ranged from 0.93 g in *Chrysanthemum x hybrid* pink to 13.40 g in *Helianthus annuus* yellow. In the Caryophyllaceae family, the longitudinal diameter ranged from 1.43 cm in *Pelargonium hortorum* red1 to 3.30 cm in *Pelargonium hortorum* fuchsia2, while the equatorial diameter ranged from 0.69 cm in *Pelargonium hortorum* pink2 to 3.91 cm in *Pelargonium hortorum* red2. The observed variations in flower weight and size can be attributed to several factors, such as the age of the plant, the nutritional composition of the plant tissues, and the characteristics of each species [25]. Genome size, and even variations within the genome, have also been shown to influence flower weight through changes in cell size, the nucleus, and ploidy levels [26].

In this study, chrysanthemum cultivars had a weight range between 0.93 and 3.75 g. However, a study by other authors pointed out that the performance of flowering genotypes such as *Chrysanthemum* varies with climatic conditions, which directly affects flower weight [27]. Also, *H. rosa*-*sinensis*, a species native to tropical and subtropical regions, has flower sizes that can reach 15 cm, in agreement with the data obtained in this study (range between 7.48 and 12.15 cm) [28,29,30]. The diversity of sizes and shapes of edible flowers offers creative culinary applications. Smaller flowers can be used as delicate garnishes on dishes, while larger ones can be used as visual highlights to enhance dishes’ taste and presentation [6,18].

The flowers’ pH showed a wide variability, from acidic to alkaline values, even within the same family. An example is the family Malvaceae, which showed a pH range from 1.47 in *Malvaviscus arboreus* to 9.20 in *Hibiscus rosa*-*sinensis* yellow. In contrast, the pH was narrower in families such as Rosaceae, varying from 3.0 in *Rosa x hybrid* bid pink and red to 6.0 in *Rosa x hybrid* big yellow. These differences reflect the influence of various chemical and biological processes on the flower species, such as nutrient uptake, metal availability, and enzyme activity. Variations in pH can also be attributed to factors specific to the soil in which the flowers grew and to the adaptive strategies developed by each species [31]. On the other hand, the pH of edible flowers can vary considerably between species. For example, in a study of the species *Viola cornuta*, *Viola tricolor*, *Antirrhinum majus*, *Diantjus chinensis,* and *Tagetes patula*, it was found that the pH only increased significantly in the flowers of *T. patula* during the post-harvest period. This suggests that while some species maintain stable pH levels, others may experience changes during storage [32].

The soluble solids (°Brix) contain a mixture of sugars, organic acid, and other soluble compounds that play an essential role in plant metabolism as a source of energy and are responsible for plants’ taste and sensory quality [33,34]. In this study, flowers showed a wide range of soluble solids with concentrations comparable to the sweetness of traditional fruits such as watermelon (*Citrullus lanatus*), with values ranging from 10.43 to 13.56 °Brix [35]. Significant variation was observed among species of the same family; for example, in the Rosaceae family, soluble solids ranged from 2.06 °Brix in *Rosax hybrid* medium red to 11.00 °Brix in *Rosa x hybrid* roseta big red. On the other hand, the present study found a range of soluble solids between 2.0 and 3.9 °Brix for *Dahlia pinnata* varieties. However, another study on different *Dahlia* species reported much lower values, between 0.14 and 0.20 °Brix [36]. These differences could be due to genetic variation, environmental conditions or differences in the maturity of the flowers. One study found that edible flowers from the Lamiaceae family, particularly the *Mentheae tribe*, had a higher sugar content than the *Ocimeae tribe*. This suggests that soluble solids content can vary considerably between different botanical families and species within the same family [37].

Edible flowers are used in food for their interesting acidic flavours. In this respect, some species have been found to have high acidity levels. For example, in the family Geraniaceae, titratable acidity values ranging from 0.46% in *Pelargonium hortorum* pink to 3.21% in *Pelargonium hortorum* red-white were recorded. Thus, total titratable flower acidity, which mainly assesses organic acids, has a significant impact on edible flowers’ flavour, shelf life, and stability. Variations in titratable acidity can be attributed to factors such as the metabolic and physiological characteristics of individual plants, environmental conditions, and the developmental stage of the flower [33,34]. In this context, this study used fully developed flowers, as acidity tends to be higher at this stage. This is in line with previous studies showing an increase in acidity during flower development, as observed in an analysis of the feijoa flower (*Acca sellowiana*) [34]. Similarly, in this study, titratable acidity values between 0.12 and 0.23% were reported in *Dahlia pinnata* cultivars, whereas in previous studies on different *Dahlia* species, values between 0.4 and 0.8% were recorded [36]. Thus, edible flowers’ total soluble solids content can change significantly during post-harvest storage, generally increasing over time. This increase in soluble solids is often accompanied by a decrease in titratable acidity, which can alter the sensory profile of the flowers, particularly in terms of flavour [32].

Moisture is a crucial factor influencing the quality and longevity of flowers after harvest. Plant water content is related to growth and development and facilitates the efficient functioning of physiological processes. However, while high moisture levels are beneficial for maintaining the quality of edible flowers, they also increase their susceptibility to infection [38]. Thus, the variation in flower humidity in this study can be attributed to species-specific characteristics and are influenced by factors such as nectar evaporation, flower transpiration, flower morphology, and other physiological characteristics [39]. Other studies have reported moisture values of 74.52%, 86.98 to 88.12%, 86.15%, and 78.68% for the same species grown in Spain [12].

As regards ash content, this parameter provides valuable information on the mineral composition of plants, which can vary considerably between varieties and has implications for their use as natural antioxidants in human health [38].

### 4.2. Quantification of Total Carotenoids and Phenols and Antioxidants Activity

The total concentration of carotenoids in edible flowers varied considerably among the different species studied. Thus, in the Asteraceae family, the concentration of total carotenoids ranged from 57.65 mg β-carotene/100 g DW in *Chrysanthemum x hybrid* pink to 5154.56 mg β-carotene/100 g DW in *Helianthus annuus* yellow. In comparison, the Rosaceae family showed a range from 55.312 mg β-carotene/100 g DW in *Rosa x hybrid* mini red to 600.70 β-carotene/100 g DW in *Rosa x hybrid* mini orange. These variations can be attributed to various factors such as species genetics, growing conditions, exposure to sunlight, and environmental influences [17,18]. For example, carotenoids in Dahlia pinnata ranged from 78.64 mg/100 g DW (fuchsia1) to 931.92 mg/100 g DW (orange). However, other studies in botanical gardens in Spain reported a much lower value of 40.01 μg/g DW for yellow *Dahlia* [12]. It is also important to note that the literature reports a total carotenoid content of 162.00 ug/g fresh weight for *Hibiscus rosa*-*sinensis* grown at the Faculty of Agriculture in Cairo [40]. In contrast, the present study shows a concentration ranging from 116.47 mg/100 g DW to 2468.5 mg/100 g DW. This difference in concentration could be due to the cultivation conditions since the previous study was conducted under the ambient conditions of 35 °C and 40% relative humidity. In contrast, in this study, samples were taken from the natural habitat of Ecuador’s species at 25 °C and relative humidity between 85 and 90%.

Species with higher concentrations of total phenolics included *Hibiscus rosa*-*sinensis* red (343.84 mg GAE/g DW), *Rosa x hybrid* medium yellow (363.90 mg GAE/g DW), *Pelargonium hortorum* orange2 (523.39 mg GAE/g DW), and *Rosa x hybrid* mini red (586.37 mg GAE/g DW). These species can be considered rich sources of phenolic compounds, as phenols are widely known for their remarkable antioxidant properties and potential benefits for human health. These compounds act as potent defenders against oxidative stress and have been suggested to be essential in preventing chronic diseases such as heart disease, cancer, and neurodegenerative disorders [6].

As for phenolic compounds, the samples under study showed high concentrations of these molecules, which stand out for their antioxidant properties and act as potent defenders against oxidative stress, and are essential in the prevention of chronic diseases such as heart disease, cancer, and neurodegenerative disorders. In this context, in *Hibiscus rosa*-*sinensis*, the concentration of phenolic compounds ranged from 152.0 mg GAE/g DW (red 2) to 353.8 mg GAE/g DW (yellow). These values were higher than those reported by other authors, who reported a concentration of 61.45 mg/100 g when extracted with methanol and 59.3 mg/100 DW when extracted with ethanol [41]. In addition, another study reported concentrations of total phenolics in *Hibiscus rosa*-*sinensis* grown in Cairo using different solvents. The concentrations ranged from 186.17 mg/100 g FW, 235.77 mg/100 g FW, to 281.23 mg/100 g FW using absolute ethanol, water, and 80% ethanol, respectively [40]. The variation in total phenolic content observed between species can be attributed to genetic and environmental factors such as soil type, nutrient availability, exposure to sunlight, and other environmental conditions, as suggested by other authors [12,18].

Regarding antioxidant activity, the high concentration found in the flowers in this study indicate the presence of bioactive compounds such as carotenoids and phenols, which can neutralise free radicals and prevent oxidation [42,43]. Phenolic compounds are mainly known to be potent antioxidants [44,45]. However, differences in antioxidant activity observed between species and within the same genus, such as in the Rosaceae family, where the antioxidant activity ranged from 276.79 mmol ET/100 g DW in *Rosa x hybrid* to 775.30 mmol ET/100 g DW in *Rosa canina*, could be due to differences in the concentrations of antioxidant bioactive compounds. These differences reflect the plants’ genetic characteristics and external influences, such as growing conditions and environmental factors [46]. For example, the antioxidant activity of *Hibiscus rosa*-*sinensis*, evaluated by the DPPH assay using water, 80% ethanol, and absolute ethanol as solvents at concentrations of 500, 1000, and 2000 mg/L, showed an inhibition range between 2.78% and 80.78% [38]. These values are related to the data obtained in this study, which ranged from 52.78% (orange2) to 88.02% (orange1). They were also related to another study which reported 75% inhibition in a methanolic extract [41].

### 4.3. General Statistical Analysis

The complexity of classifying flowers according to their characteristics makes it difficult to analyse them individually. Therefore, the present study applied a statistical analysis of physicochemical parameters, carotenoid and total phenolic content, and antioxidant activity of the 93 species evaluated. A correlation analysis approach using networks and principal components was used.

The results showed a positive correlation between colour coordinate b* and total carotenoids, coordinate a* and total phenolics, and pH and antioxidant activity. In contrast, a negative correlation was observed between weight and titratable acidity, colour intensity and a* and b* coordinates, and between a* and pH and antioxidant activity.

Figure 2B shows the principal component analysis (PCA), where the first component (Dim1) explains 19.1% of the variance and the second (Dim2) 15.5%. The PCA shows the correlation between total phenolics, antioxidant activity, a* coordinate, and percentage inhibition. The known relationship between weight and size and a positive correlation between pH and ash content were also confirmed.

These results are consistent with previous studies that reported a direct relationship between the a* colour coordinate and total phenolics [47], between the b* colour coordinate and carotenoids [48], and an inverse correlation between colour intensity and its polar coordinates [49]. In addition, studies have shown a correlation between antioxidant activity and total phenolic compounds [47].

### 4.4. Bioactive Compound Profiles

Organic acids are important in activating and mobilising essential nutrients, mainly by modifying soil chemistry and enhancing microbial activity, promoting plant growth and improving edible flower’s nutritional profile [50]. Thus, in this study, all species showed concentrations of organic acids, with citric acid having the highest concentration in *H. rosa*-*sinensis* red1. These levels suggest considerable interspecies variability in the accumulation of these compounds.

In addition to their role in plant physiology, organic acids influence foods’ taste, nutritional value, and shelf life. They are found in various edible fruits, vegetables, and flowers, each with a unique profile that contributes to distinctive sensory characteristics. For example, tartaric acid, which contributes to the acidic taste of many flowers, ranged from 18.2 mg/g DW in *H. rosa*-*sinensis* (45) to 113.8 mg/g DW in *Canna indica* (12). Similarly, malic acid, a compound involved in regulating cell metabolism, showed concentrations ranging from 6.2 mg/g DW in *H. rosa*-*sinensis* (45) to 119.4 mg/g DW in *Pelargonium hortorum* (19). Citric acid, known for its antioxidant and preservative properties, was the predominant organic acid, with concentrations ranging from 11.0 mg/g DW in *Canna indica* to 991.4 mg/g DW in *H. rosa*-*sinensis* (49). This agrees with previous studies on species such as *Theobroma speciosum*, which also showed a predominance of citric acid [51].

From a nutritional point of view, organic acids are essential for energy production, acting as intermediates in critical metabolic cycles such as the Krebs cycle. They also support regulating metabolism and immune health and benefit heart function. An essential aspect of organic acids in food is their ability to inhibit bacterial growth. These compounds can alter bacterial homeostasis and enzyme activity, making them natural allies in food preservation and the fight against foodborne pathogens [52]. In this sense, species such as *Hibiscus rosa*-*sinensis*, which have high concentrations of citric acid, become an essential resource against pathogenic micro-organisms.

On the other hand, phenolic compounds are important secondary metabolites in plants, widely distributed as phenolic acids, flavonoids, and glycosides. These bioactive molecules are involved in plant defence and are associated with numerous benefits for human health, such as anti-inflammatory and antimicrobial properties. Their presence and concentration in edible flowers can vary significantly depending on intrinsic factors such as plant genetics and stage of development, as well as environmental conditions such as soil type, altitude, and water stress [6,12]. The phenolic concentration of the selected flowers, as the sum of the individual compounds, ranged from 175.0 mg/100 g DW in *H. annuus* to 2827.4 mg/100 g DW in *T. patula*.

Among the most abundant phenolic compounds in the selected flowers, gallic acid, vanillic acid, *p*-coumaric acid, *m*-coumaric acid, syringic acid, chlorogenic acid, caffeic acid, naringenin, ferulic acid, 4-hydroxybenzoic acid, kaempferol, quercetin glycoside, and quercetin, as suggested by other authors [12]. Among these, gallic acid, known for its potent antioxidant capacity and anticarcinogenic potential, showed a moderately low concentration in the species studied, with *Pelargonium hortorum* (19) having the highest value (17.2 mg/100 g DW). 4-hydroxybenzoic acid, known for its antimicrobial and antioxidant properties, was dominant in *P. hortorum* with a 936.2 mg/100 g DW concentration. This acid is particularly valued for its ability to act as a natural preservative, inhibiting the growth of pathogens and protecting cells from oxidative damage.

Rutin, a flavonoid known for its anti-inflammatory and cardioprotective properties, was abundant in *Hibiscus rosa*-*sinensis* (854.3 mg/100 g DW). Similarly, kaempferol, a flavonoid with well-documented anti-cancer effects, reached its highest concentration in *T. patula* (971.9 mg/100 g DW). Quercetin and its derivatives, known for their potent antioxidant and anti-inflammatory effects, were highly concentrated in *C. x hybrid* (958.8 mg/100 g DW) and *T. patula* (936.2 mg/100 g DW). Chlorogenic and caffeic acids, known for their ability to regulate lipid and glucose metabolism as well as their antioxidant activity, were found in moderate concentrations in *H. rosa*-*sinensis* (1.2 mg/100 g DW chlorogenic acid and 3.6 mg/100 g DW caffeic acid).

Carotenoids are a diverse group of natural lipophilic pigments found in photosynthetic organisms such as plants, algae, and some bacteria. These compounds, responsible for colours ranging from yellow to red, are essential in photosynthesis and photoprotection [46]. The carotenoid concentration of the selected flowers, as the sum of the individual compounds, ranged from 2.5 mg/100 g DW (49 *H. rosa*-*sinensis*) to 2043.3 mg/100 g DW (*T. patula* orange). The latter stands out as a significant source of carotenoids, followed by *H. annuus* yellow (992.5 mg/100 g DW) and *C. indica* red (703.1 mg/100 g DW).

Among the most abundant carotenoids in the selected flowers are α-carotene, β-carotene, β-cryptoxanthin, violaxanthin, zeaxanthin, lutein, and zeinoxanthin, all of which have various bioactive properties, as suggested by other authors [12]. Thus, orange is exceptionally high in α-carotene (989.5 mg/100 g DW) and β-carotene (601.2 mg/100 g DW), while *H. annuus* yellow also has a high concentration of α-carotene (604.0 mg/100 g DW). These two carotenoids are precursors of vitamin A, an essential nutrient for vision, immunity, and skin integrity [12,18]. Another carotenoid with provitamin A activity, β-cryptoxanthin, was found in high concentrations in *T. patula* (34.2 mg/100 g DW) and to a lesser extent in *H. rosa*-*sinensis* yellow (12.1 mg/100 g DW).

The xanthophylls violaxanthin and zeaxanthin are essential for their protective effects against age-related macular degeneration [47]. Thus, violaxanthin was found in *H. annuus* yellow (59.5 mg/100 g DW) and zeaxanthin, abundant in *T. patula* orange (149.5 mg/100 g DW), and lutein was found in significant amounts in *H. annuus* yellow (300.9 mg/100 g DW) and *T. patula* orange (219.3 mg/100 g DW).

### 4.5. Antimicrobial Activity

The evaluation of the flower extracts’ antimicrobial activity included antibacterial and antifungal assays. The bacterial strains used were *Escherichia coli*, *Staphylococcus aureus*, *Pseudomonas aeruginosa*, *Streptococcus mutans*, and the pathogenic fungi *Candida albicans* and *Candida tropicalis*. These micro-organisms cause various human infections, including urinary, respiratory, skin, and oral infections. The ability to inhibit the growth of micro-organisms is exciting for the health sector, as conventional treatments have been found to be ineffective against bacteria and fungi [53].

*Escherichia coli*, a Gram-negative bacterium commonly associated with urinary and intestinal infections [54], showed inhibition against extracts of *T. patula*, *Hibiscus rosa*-*sinensis* (orange1), *P. hortorum*, and *Rosa x hybrid*. In this regard, studies have shown that gallic acid significantly inhibits the growth of *E. coli* by altering cell morphology and reducing glucose consumption [55]. Similarly, chlorogenic acid has been identified as an effective agent in reducing virulence factors and biofilm formation [56]. According to the results presented in Table 3, some of the species mentioned contain concentrations of these phenolic compounds, suggesting that the observed antimicrobial activity could be related to the presence of these bioactives. Further studies have shown that organic acids, such as citric acid, in combination with hot water, have a potent bactericidal effect against *E. coli* biofilms [57]. This suggests that the *E. coli* inhibitory activity observed in the selected species could be influenced by phenols and the presence of organic acids, as suggested by a study on 17 Iranian *Chrysanthemum morifolium* cultivars [58]. Still, in this study, there was no inhibition of *E. coli* by this flower extract.

*Staphylococcus aureus*, a Gram-positive bacterium responsible for skin, respiratory, and systemic infections, has shown increasing antibiotic resistance, making it difficult to treat in the population. In this study, most of the extracts tested, except *C. indica*, showed inhibitory activity against *S. aureus* (Table 5). This inhibition can be attributed to the presence of bioactive compounds such as kaempferol, quercetin, *p*-coumaric acid, caffeic acid, gallic acid, ferulic acid, chlorogenic acid, and naringenin, all of which have been shown to have antimicrobial activity against *S. aureus* [53,59]. Furthermore, the results reported in this study agreement with other authors who reported inhibition in an aqueous extract of *H. rosa-sinensis* against *S. aureus* and *P. aeruginosa* [59] and *T. patula* against *E. coli* and *S. aureus*.

In addition, the selected extracts contained organic acids, mainly citric acid, which contributes to the reduction of pH, creating an unfavourable acidic environment for the growth of *S. aureus*. These results are consistent with previous studies suggesting that a reduced pH effectively inhibits the growth of this bacterium [60].

*Pseudomonas aeruginosa*, an opportunistic pathogen highly resistant to multiple treatments, is associated with severe infections in immunocompromised patients. Most selected extracts showed antimicrobial activity against *P. aeruginosa* in this study, except for *H. annuus* and *H. rosa*-*sinensis* (yellow). These species contain various concentrations of phenolic compounds, such as naringin, rutin, chlorogenic acid, ferulic acid, *p*-coumaric acid, quercetin, and gallic acid, which have shown antimicrobial activity against *P. aeruginosa* [61,62].

Regarding *Streptococcus mutans*, a bacterium involved in forming dental caries [63], excellent inhibition was observed by extracts of *H. annuus*, *T. patula*, *H. rosa*-*sinensis* (orange1 and red), and *P. hortorum*. Some of these species also showed high concentrations of lutein, a xanthophyll which, like zeaxanthin, has been shown to have inhibitory effects against *P. aeruginosa* [61].

Regarding antifungal activity, *Candida albicans*, a fungus responsible for infections such as candidiasis, showed inhibition against *C. x hybrid* extracts, suggesting the presence of bioactive compounds capable of exerting an antifungal effect. On the other hand, *Candida tropicalis*, an opportunistic fungal pathogen that mainly affects immunocompromised patients, was inhibited by *P. hortorum* extracts.

Previous studies have shown that caffeic acid has remarkable antifungal activity against *C. albicans* and *C. tropicalis* [64]; however, no such compounds were detected in *C. x hybrid* in this study. This result suggests that other bioactive compounds in *C. x hybrid*, possibly flavonoids or terpenoids, could contribute to the observed antifungal activity.

In this context, most of the selected species showed antibacterial activity, except *C. indica*, which showed no inhibitory effect against the micro-organisms tested. This finding points to the remarkable variability in the chemical composition of flower extracts among different species, which directly impacts their antimicrobial efficacy. In contrast, antifungal activity was restricted to a few species, with *C. x hybrid* and *P. hortorum* being the most effective. This suggests that the bioactive compounds responsible for fungal inhibition may be more specific or present at lower concentrations in the other species tested.

In the case of *Hibiscus rosa*-*sinensis*, the study revealed significant differences in antimicrobial activity between the different varieties. Against *Staphylococcus aureus*, the orange1 variety showed an inhibition index of 0.5, the yellow variety showed an index of 0.4, and the red variety showed no inhibitory activity. For *Escherichia coli*, *H. rosa-sinensis* orange1 showed an inhibition index of 0.3. These results agree with previous studies reporting inhibition zones of 14 mm for *Staphylococcus* sp. and 13 mm for *E. coli* in ethanolic extracts [41].

This pattern suggests that the bioactivity of flower extracts is influenced not only by the plant variety but also by the extraction method used. The variability in the phenolic and flavonoid profile between different *H. rosa*-*sinensis* cultivars could be a determining factor in their antimicrobial capacity. This indicates the importance of selecting the appropriate cultivar and the optimal extraction conditions to maximise flower extracts’ bioactive and antimicrobial potential.

### 4.6. Specific Statistical Analysis

The biological activities of plants are closely linked to the presence of their primary and secondary metabolites. However, analysing each molecule separately does not provide insight into the complex interactions that benefit human health. In this context, Figure 3A shows a network of correlations between antioxidant activity, phenolic profiles, carotenoids, organic acids, and antimicrobial activity against various micro-organisms. Direct relationships were found between *Escherichia coli* with *Pseudomonas aeruginosa* and *Candida tropicalis* and between *Pseudomonas aeruginosa* and *Staphylococcus aureus*. Associations were also observed between chlorogenic acid and naringin; caffeic acid and vanillic acid; naringin and rutin; ferulic acid with violaxanthin and luteolin; naringin and citric acid; lutein and α-carotene; β-carotene and zeaxanthin; zeinoxanthin and zeaxanthin; lutein and β-carotene; and between kaempferol and quercetin. In addition, a significant correlation was found between percentage inhibition and malic acid, and between 4-hydroxybenzoic acid, *p*-coumaric acid, and gallic acid. An inverse relationship was found between antioxidant activity and citric acid, consistent with previous studies suggesting that citric acid favours phenolic biosynthesis. Plants consume citric acid to produce higher phenolic compounds and flavonoids [65].

The correlation between *E. coli* and *P. aeruginosa* reflects a complex interaction with antagonistic and cooperative elements. Although *E. coli* is a common inhabitant of the gut, it can inhibit colonisation by *P. aeruginosa*, which is relevant given the pathogenic capacity of the latter in the lungs and wounds. This antagonism is partly attributed to the production of lactic acid by *E. coli*, which inhibits the growth of *P. aeruginosa*, especially in a high-sugar diet [66]. On the other hand, the interaction between *E. coli* and *C. tropicalis* influences biofilm formation and microbial dynamics. Lipopolysaccharide from *E. coli* has been shown to modulate *Candida* biofilm formation. Studies show a significant reduction in *C. tropicalis* colony-forming units when cultured with *E. coli*. However, an increase in *E. coli* cells is also observed after 24 h of co-culture [67]. Similarly, the interaction between *P. aeruginosa* and *S. aureus* has both competitive and synergistic dynamics. *P. aeruginosa* can antagonise *S. aureus* through cell lysis, facilitating biofilm formation and survival under co-culture conditions [68]. The combination of naringin and β-carotene has been found to stimulate the expression of genes related to glucose metabolism, promote thermogenesis, and improve insulin sensitivity in adipocytes [69]. In addition, the levels of phenolics and carotenoids vary considerably between plant sources. Flowers with high levels of carotenoids often have reduced levels of phenolics, suggesting a complex relationship between these compounds in natural matrices [12].

The principal component analysis shown in Figure 3B, which shows the distribution of bioactive compounds and micro-organisms, showed that the first principal component explained 25.1% of the variability, while the second explained 21.4%. Inhibition of antioxidant activity and quercetin glycoside are strongly associated with Dim2, while *Candida albicans* and malic acid are mainly associated with Dim1. This graph also shows close relationships between different molecules and micro-organisms. For example, there is a strong correlation between quercetin, zeinoxanthin, zeaxanthin, β-carotene, α-carotene, lutein, luteolin, kaempferol, and violaxanthin, as well as rutin, naringin, chlorogenic acid, and caffeic acid. Significant associations were also found between malic acid, 4-hydroxybenzoic acid, gallic acid, *p*-coumaric acid, and *C. tropicalis* activity. The observed correlations between carotenoids and phenolic compounds reflect specific metabolic pathways for the biosynthesis of these molecules [70].

## 5. Conclusions

Edible flowers have been used since ancient times, but their potential in human health, nutraceutical, and pharmaceutical applications is still an emerging area of research. This physicochemical and bioactive study showed significant variations among the species studied in their structural characteristics and chemical composition. In terms of physicochemical properties, the flowers showed great diversity in parameters such as pH, ranging from highly acidic values (pH 0.8 in *Pelargonium hortorum* pink-white2) to strongly alkaline values (pH 13 in *Raphanus rapahanistrum*, *Dianthus chinensis* red, *Pelargonium hortorum* red2, and *Antirrhinum majus* red). The soluble solids content was remarkably high (11 °Brix) in species such as *Raphanus raphanistrum* and *Rosa x hybrid* variety rosette big red. In contrast, species such as *Dianthus chinensis* red were characterised by high titratable acidity (4.69%). Total carotenoids ranged from 32.28 mg/100 g DW in *Pelargonium hortorum* white1 to 5745.28 mg/100 g DW in *Calendula officinalis*. In comparison, total phenolics ranged from 16.49 mg GAE/g DW in *Chamaemelun nobile* to 586.37 mg GAE/g DW in *Rosa x hybrid* variety mini red. High levels of antioxidant activity were also observed with species such as *Citrus x aurantifolia* (100% inhibition), *Dahlia pinnata* orange (90.86%), and *Pelargonium hortorum* pink (90.91%). On the other hand, some flowers (*Chrysanthemum x hybrid* orange, *Helianthus annuus* yellow, *Tagetes patula* orange, *Canna indica* red, and *Hibiscus rosa*-*sinensis* orange1 and yellow) showed significant concentrations of total carotenoids. In contrast, *Pelargonium hortorum* orange2, *Hibiscus rosa*-*sinensis* red1, and *Rosa x hybrid* variety medium yellow showed high concentrations of phenolics. Thus, these species showed interesting concentrations of organic compounds, with citric acid being the predominant one, ranging from 8.9 mg/g DW in *H. annuus* to 991.4 mg/g DW in *H. rosa*-*sinensis* red1. The phenolic profile showed the presence of gallic acid, vanillin, *p*-coumaric acid, *m*-coumaric acid, syringic acid, chlorogenic acid, caffeic acid, ferulic acid, 4-hydroxybenzoic acid, as well as naringin, rutin, kaempferol, quercetin glucoside, and quercetin, with significant values for the last three compounds. In the case of carotenoid profiles, the presence of α-carotene, β-carotene, β-cryptoxanthin, violaxanthin, zeaxanthin, lutein, and zeinoxanthin was observed, with significant concentrations of the first two provitamin carotenoids. In terms of antimicrobial activity, flowers such as *T. patula* orange with a high concentration of carotenoids and *P. hortorum* orange2 showed effective inhibition against pathogenic bacteria such as *Escherichia coli*, *Staphylococcus aureus*, *Pseudomonas aeruginosa*, and *Streptococcus mutans*. In addition, *C. x hybrid* orange and *P. hortorum* orange2 inhibited *Candida albicans*, although the latter flower inhibited *Candida tropicalis*.

## Figures and Tables

**Table 1:** Geographical distribution of flowers under study.

N°	Family	Species	Sampling Location		Altitude (masl)
1	Apiaceae	*Anethum graveolens* (yellow)	1.0°18.0′52.0″ S	78.0°31.0′46.0″ W	2350
2	Asteraceae	*Chrysanthemum x hybrid* (pink)	2.0°53.0′11.0″ S	78.0°59.0′23.0″ W	2728
3		*Chrysanthemum x hybrid* (orange)	2.0°53.0′11.0″ S	78.0°59.0′23.0″ W	2728
4		*Chrysanthemum x hybrid* (yellow)	2.0°53.0′11.0″ S	78.0°59.0′23.0″ W	2728
5		*Chrysanthemum x hybrid* (yellow-double)	1.0°23.0′43.0″ S	78.0°26.0′18.0″ W	1600
6		*Helianthus annuus* (yellow)	0.0°10.0′58.7″ S	78.0°22.0′59.9″ W	2339
7		*Tagetes patula* (orange)	0.0°9.0′0.0″ S	78.0°25.0′60.0″ W	2600
8		*Tagetes patula* (yellow)	0.0°09.0′0.0″ S	78.0°25.0′60.0″ W	2600
9	Begoniaceae	*Begonia doblet* (pink)	0.0°10.0′58.7″ S	78.0°22.0′59.5″ W	2339
10	Brasicaceae	*Raphanus raphanistrum* (pink)	0.0°09.0′0.0″ S	78.0°25.0′60.0″ W	2600
11	Cannaceae	*Canna indica* (red-double)	0.0°11.0′19.0″ S	78.0°23.0′46.0″ W	2360
12		*Canna indica* (red)	0.0°05.0′14.0″ S	78.0°26.0′59.9″ W	2610
13		*Canna indica* (yellow-orange)	0.0°11.0′19.0″ S	78.0°23.0′46.0″ W	2360
14	Caryophyllaceae	*Dianthus chinensis* (pink)	0.0°09.0′0.0″ S	78.0°25.0′60.0″ W	2600
15		*Dianthus chinensis* (red)	0.0°09.0′0.0″ S	78.0°25.0′60.0″ W	2600
16		*Pelargonium hortorum* (fuchsia1)	0.0°09.0′0.0″ S	78.0°25.0′60.0″ W	2600
17		*Pelargonium hortorum* (fuchsia2)	0.0°20.0′4.8″ S	78.0°33.0′57.7″ W	3121
18		*Pelargonium hortorum* (orange1)	0.0°09.0′0.0″ S	78.0°25.0′60.0″ W	2600
19		*Pelargonium hortorum* (orange2)	0.0°20.0′4.8″ S	78.0°33.0′57.7″ W	3121
20		*Pelargonium hortorum* (pink1)	0.0°09.0′0.0″ S	78.0°25.0′60.0″ W	2600
21		*Pelargonium hortorum* (pink2)	0.0°09.0′0.0″ S	78.0°25.0′60.0″ W	2600
22		*Pelargonium hortorum* (pink3)	0.0°09.0′0.0″ S	78.0°25.0′60.0″ W	2600
23		*Pelargonium hortorum* (pink4)	0.0°20.0′4.8″ S	78.0°33.0′57.7″ W	3121
24		*Pelargonium hortorum* (pink-fuchsia)	0.0°09.0′0.0″ S	78.0°25.0′60.0″ W	2600
25		*Pelargonium hortorum* (pink-white1)	0.0°09.0′0.0″ S	78.0°25.0′60.0″ W	2600
26		*Pelargonium hortorum* (pink-white2)	0.0°09.0′0.0″ S	78.0°25.0′60.0″ W	2600
27		*Pelargonium hortorum* (red1)	0.0°09.0′0.0″ S	78.0°25.0′60.0″ W	2600
28		*Pelargonium hortorum* (red2)	0.0°09.0′0.0″ S	78.0°25.0′60.0″ W	2600
29		*Pelargonium hortorum* (white1)	0.0°09.0′0.0″ S	78.0°25.0′60.0″ W	2600
30		*Pelargonium hortorum* (white2)	0.0°20.0′4.8″ S	78.0°33.0′57.7″ W	3121
31	Compositae	*Calendula officinalis*(yellow)	1.0°23.0′43.0″ S	78.0°26.0′18.0″ W	1600
32		*Chamaemelun nobile* (white)	1.0°11.0′51.5″ S	78.0°32.0′9.1″ W	2908
33		*Dahlia pinnata* (fuchsia1)	1.0°19.0′19.0″ S	78.0°30.0′46.0″ W	2360
34		*Dahlia pinnata* (fuchsia2)	1.0°23.0′43.0″ S	78.0°26.0′18.0″ W	1600
35		*Dahlia pinnata* (orange)	1.0°23.0′43.0″ S	78.0°26.0′18.0″ W	1600
36		*Dahlia pinnata* (red)	1.0°23.0′43.0″ S	78.0°26.0′18.0″ W	1600
37	Fabaceae	*Trifolium repens* (white)	1.0°23.0′43.0″ S	78.0°26.0′18.0″ W	1600
38	Geraniaceae	*Pelargonium hortorum* (fuchsia1)	0.0°10.0′58.7″ S	78.0°22.0′59.5″ W	2339
39		*Pelargonium hortorum* (fuchsia2)	0.0°11.0′12.0″ S	78.0°22.0′59.9″ W	2339
40		*Pelargonium hortorum* (pink)	0.0°11.0′14.8″ S	78.0°22.0′55.1″ W	2339
41		*Pelargonium hortorum* (red1)	0.0°11.0′19.0″ S	78.0°23.0′46.0″ W	2360
42		*Pelargonium hortorum* (red2)	0.0°10.0′58.7″ S	78.0°22.0′59.5″ W	2339
43		*Pelargonium hortorum* (red-white)	0.0°11.0′19.0″ S	78.0°23.0′46.0″ W	2342
44	Lamiaceae	*Salvia microphylla* (blue)	0.0°11.0′19.0″ S	78.0°23.0′46.0″ W	2360
45	Malvaceae	*Hibiscus rosa-sinensis* (orange1)	0.0°12.0′28.5″ N	78.0°29.0′14.8″ W	2738
46		*Hibiscus rosa-sinensis* (orange2)	0.0°12.0′28.5″ N	78.0°29.0′14.8″ W	2738
47		*Hibiscus rosa-sinensis* (pink 1)	0.0°12.0′28.5″ N	78.0°29.0′14.8″ W	2738
48		*Hibiscus rosa-sinensis* (pink 2)	0.0°19.0′0.0″ S	78.0°22.0′59.9″ W	2644
49		*Hibiscus rosa-sinensis* (red 1)	0.0°12.0′28.5″ N	78.0°29.0′14.8″ W	2738
50		*Hibiscus rosa-sinensis* (red 2)	0.0°53.0′0.9″ N	79.0°47.0′59.9″ W	25
51		*Hibiscus rosa-sinensis* (yellow)	0.0°12.0′28.5″ N	78.0°29.0′14.8″ W	2738
52		*Malvaviscus arboreus* (red)	1.0°23.0′35.0″ S	78.0°26.0′47.0″ W	1850
53	Nyctaginaceae	*Mirabilis jalapa* (fuchsia)	0.0°6.7′31.0″ S	78.0°27.0′39.6″ W	2681
54	Onagraceae	*Fuchsia magellanica* (pink)	0.0°11.0′19.0″ S	78.0°23.0′46.0″ W	2360
55	Plantaginaceae	*Antirrhinum majus* (red)	0.0°9.0′0.0″ S	78.0°25.0′60.0″ W	2600
56		*Antirrhinum majus* (yellow)	0.0°11.0′1.4″ S	78.0°22.0′59.9″ W	2339
57	Rosaceae	*Rosa banksiae* (white)	0.0°22.6′58.5″ S	78.0°33.2′15.0″ W	2945
58		*Rosa canina* (pink)	1.0°23.0′43.0″ S	78.0°26.0′18.0″ W	1600
59		*Rosa damascene* (pink)	0.0°15.0′0.4″ S	78.0°28.0′59.9″ W	2929
60		*Rosa x hybrid* big pink	13.0°13.0′0.0″ S	78.0°24.0′0.0″ W	2324
61		*Rosa x hybrid* big red	13.0°13.0′0.0″ S	78.0°24.0′0.0″ W	2325
62		*Rosa x hybrid big white*	13.0°13.0′0.0″ S	78.0°24.0′0.0″ W	2325
63		*Rosa x hybrid* big yellow	13.0°13.0′0.0″ S	78.0°24.0′0.0″ W	2325
64		*Rosa x hybrid* medium (orange-yellow)	0.0°9.0′0.0″ S	78.0°25.0′60.0″ W	2600
65		*Rosa x hybrid* medium orange	0.0°9.0′0.0″ S	78.0°25.0′60.0″ W	2600
66		*Rosa x hybrid* medium pink	0.0°9.0′0.0″ S	78.0°25.0′60.0″ W	2600
67		*Rosa x hybrid* medium purple	0.0°9.0′0.0″ S	78.0°25.0′60.0″ W	2600
68		*Rosa x hybrid* medium red	0.0°9.0′0.0″ S	78.0°25.0′60.0″ W	2600
69		*Rosa x hybrid* medium white	0.0°9.0′0.0″ S	78.0°25.0′60.0″ W	2600
70		*Rosa x hybrid* medium yellow	0.0°9.0′0.0″ S	78.0°25.0′60.0″ W	2600
71		*Rosa x hybrid* mini red	0.0°26.0′0.1″ S	78.0°32.0′0.6″ W	2750
72		*Rosa x hybrid* mini orange	0.0°26.0′0.1″ S	78.0°32.0′0.6″ W	2750
73		*Rosa x hybrid* mini red	0.0°26.0′0.1″ S	78.0°32.0′0.6″ W	2750
74		*Rosa x hybrid* mini yellow	0.0°26.0′0.1″ S	78.0°32.0′0.6″ W	2750
75		*Rosa x hybrid* roseta medium orange-yellow	0.0°9.0′0.0″ S	78.0°25.0′60.0″ W	2600
76		*Rosa x hybrid* roseta medium orange	0.0°9.0′0.0″ S	78.0°25.0′60.0″ W	2600
77		*Rosa x hybrid* roseta medium purple	0.0°9.0′0.0″ S	78.0°25.0′60.0″ W	2600
78		*Rosa x hybrid* roseta medium pink	0.0°9.0′0.0″ S	78.0°25.0′60.0″ W	2600
79		*Rosa x hybrid* roseta medium red	0.0°9.0′0.0″ S	78.0°25.0′60.0″ W	2600
80		*Rosa x hybrid* roseta mini pink	0.0°9.0′0.0″ S	78.0°25.0′60.0″ W	2600
81		*Rosa x hybrid* roseta mini white	0.0°9.0′0.0″ S	78.0°25.0′60.0″ W	2600
82		*Rosa x hybrid* roseta big purple	0.0°10.0′58.7″ S	78.0°22.0′59.5″ W	2339
83		*Rosa x hybrid* roseta big red	0.0°11.0′19.0″ S	78.0°23.0′46.0″ W	2360
84		*Rosa x hybrid* rosetabig pink-white	0.0°11.0′19.0″ S	78.0°23.0′46.0″ W	2360
85		*Rosa x hybrid* roseta big yellow	0.0°11.0′19.0″ S	78.0°23.0′46.0″ W	2360
86	Rutaceae	*Citrus x aurantifolia* (white)	0.0°11.0′19.0″ S	78.0°23.0′46.0″ W	2360
87		*Ruda chalepensis* (yellow)	0.0°11.0′19.0″ S	78.0°23.0′46.0″ W	2360
88	Verbenaceae	*Aloysia citriodora* (fuchsia)	0.0°6.0′28.3″ S	78.0°26.0′51.9″ W	2654
89		*Lantana camara* multicolor	0.0°11.0′19.0″ S	78.0°23.0′46.0″ W	2360
90		*Lantana viburnoides* (red)	0.0°12.0′3.9″ S	78.0°23.0′45.9″ W	2359
91		*Lantana viburnoides* (red-orange)	0.0°12.0′19.8″ S	78.0°22.0′59.9″ W	2359
92		*Lantana viburnoides* (yellow)	0.0°12.0′3.9″ S	78.0°23.0′45.9″ W	2359
93		*Lantana viburnoides* (white)	0.0°12.0′3.9″ S	78.0°23.0′45.9″ W	2359

**Table 2:** Average values of the physicochemical characterisation of the flowers under study.

N°	Flower Extracts	Extract Concentration (mg/mL)
3	*C. x hybrid* (orange)	521.2
6	*H. annuus* (yellow)	102.2
7	*T. patula* (orange)	229.3
12	*C. indica* (red)	395.7
45	*H. rosa-sinensis* (orange1)	255.6
51	*H. rosa-sinensis* (yellow)	424.6
89	*L. camara*	128.2
19	*P. hortorum* (orange2)	136.5
49	*H. rosa-sinensis* (red1)	134.0
70	*Rosa x hybrid* medium red	218.5

**Table 3:** Average values of carotenoids and phenols and the antioxidant activity of the flowers under study.

N°	Family	Scientific Name	Total Carotenoids (mg β-Carotene/100 g DW)			Total Phenols (mg GAE/g DW)			% Inhibition ABTS^•+^			Antioxidant Activity (TEmmol/100 g DW)		
1	Apiaceae	*Anethum graveolens* (yellow)	467.62	±	1.11	254.66	±	17.10	34.67	±	0.28	277.58	±	2.53
2	Asteraceae	*Chrysanthemum x hybrid* (pink)	57.65	±	8.24	245.36	±	2.24	86.91	±	0.35	676.32	±	2.99
3		*Chrysanthemum x hybrid* (orange)	1103.46	±	5.13	155.25	±	0.73	38.73	±	0.80	209.54	±	41.89
4		*Chrysanthemum x hybrid* (yellow)	514.56	±	2.15	100.89	±	3.06	55.67	±	1.23	408.15	±	10.58
5		*Chrysanthemum x hybrid* (yellow-double)	426.39	±	7.41	199.09	±	4.02	59.32	±	1.07	504.99	±	9.77
6		*Helianthus annuus* (yellow)	5154.64	±	0.51	77.42	±	0.97	68.47	±	0.88	463.45	±	6.98
7		*Tagetes patula* (orange)	2057.79	±	3.66	194.84	±	15.99	83.77	±	0. 73	623.65	±	6.55
8		*Tagetes patula* (yellow)	500.62	±	3.05	303.61	±	2.39	83.89	±	0.39	624.71	±	3.51
9	Begoniaceae	*Begonia doblet* (pink)	194.69	±	5.33	275.17	±	1.19	41.35	±	3.63	342.91	±	33.21
10	Brasicaceae	*Raphanus raphanistrum* (pink)	270.83	±	22.53	278.38	±	13.64	80.42	±	0.74	524.83	±	5.34
11	Cannaceae	*Canna indica* (red-double)	78.86	±	11.03	261.56	±	10.66	77.01	±	2.61	661.55	±	24.15
12		*Canna indica* (red)	1223.51	±	5.33	250.09	±	16.09	53.80	±	6.65	451.46	±	63.01
13		*Canna indica* (yellow-orange)	1021.44	±	8.54	115.21	±	5.01	58.89	±	2.64	491.29	±	23.58
14	Caryophyllaceae	*Dianthus chinensis* (pink)	49.05	±	3.24	248.57	±	11.71	70.56	±	1.12	604.40	±	10.15
15		*Dianthus chinensis* (red)	75.90	±	5.85	179.63	±	4.53	81.62	±	0.67	523.76	±	3.41
16		*Pelargonium hortorum* (fuchsia1)	124.56	±	2.32	274.22	±	11.13	83.15	±	1.32	544.64	±	9.60
17		*Pelargonium hortorum* (fuchsia2)	94.16	±	1.05	286.11	±	4.20	85.67	±	0.71	530.57	±	5.06
18		*Pelargonium hortorum* (orange1)	144.54	±	1.37	331.34	±	8.52	79.24	±	0.49	516.28	±	3.52
19		*Pelargonium hortorum* (orange2)	156.30	±	3.58	525.39	±	3.94	85.99	±	0.19	532.90	±	1.37
20		*Pelargonium hortorum* (pink1)	95.01	±	3.06	218.09	±	4.78	86.41	±	0.48	504.54	±	3.22
21		*Pelargonium hortorum* (pink2)	202.73	±	2.54	281.33	±	9.29	83.22	±	1.17	545.13	±	8.48
22		*Pelargonium hortorum* (pink3)	170.33	±	0.71	193.74	±	14.01	82.42	±	0.53	539.40	±	3.83
23		*Pelargonium hortorum* (pink4)	81.18	±	1.38	302.90	±	2.11	86.14	±	0.41	427.52	±	9.38
24		*Pelargonium hortorum* (pink-fuchsia)	67.84	±	0.82	226.40	±	1.17	83.22	±	0.73	545.13	±	5.32
25		*Pelargonium hortorum* (pink-white1)	113.10	±	1.21	279.99	±	11.98	84.95	±	0.59	494.80	±	3.95
26		*Pelargonium hortorum* (pink-white2)	80.71	±	5.43	334.18	±	11.94	83.75	±	0.59	549.01	±	4.27
27		*Pelargonium hortorum* (red1)	88.19	±	9.06	110.74	±	0.33	84.38	±	0.16	442.75	±	94.64
28		*Pelargonium hortorum* (red2)	135.12	±	0.64	314.14	±	7.65	80.74	±	0.40	527.16	±	2.89
29		*Pelargonium hortorum* (white1)	32.38	±	4.38	151.86	±	13.25	84.34	±	0.79	479.21	±	93.78
30		*Pelargonium hortorum* (white2)	91.15	±	0.06	134.56	±	4.21	85.45	±	0.85	529.03	±	6.06
31	Compositae	*Calendula officinalis*(yellow)	5745.28	±	58.55	290.74	±	5.87	88.88	±	2.01	771.03	±	23.90
32		*Chamaemelun nobile* (white)	63.89	±	3.86	16.49	±	0.05	4.57	±	0.41	6.01	±	3.65
33		*Dahlia pinnata* (fuchsia1)	78.64	±	0.09	280.21	±	10.59	61.71	±	0.54	523.11	±	5.86
34		*Dahlia pinnata* (fuchsia2)	98.48	±	0.91	338.60	±	22.50	75.48	±	1.21	649.61	±	6.61
35		*Dahlia pinnata* (orange)	931.92	±	6.98	327.77	±	14.01	90.86	±	0.48	796.35	±	4.39
36		*Dahlia pinnata* (red)	282.91	±	2.09	337.46	±	8.22	83.67	±	0.65	718.24	±	6.00
37	Fabaceae	*Trifolium repens* (white)	260.37	±	9.40	167.76	±	11.06	81.43	±	0.95	554.16	±	7.25
38	Geraniaceae	*Pelargonium hortorum* (fuchsia1)	131.47	±	0.39	311.74	±	1.92	79.88	±	0.75	695.77	±	6.90
39		*Pelargonium hortorum* (fuchsia2)	94.67	±	10.59	162.11	±	10.37	86.24	±	0.68	607.50	±	5.14
40		*Pelargonium hortorum* (pink)	76.02	±	0.67	155.99	±	17.36	90.91	±	0.18	785.79	±	3.69
41		*Pelargonium hortorum* (red1)	109.83	±	2.02	262.59	±	0.40	88.71	±	0.42	776.60	±	3.88
42		*Pelargonium hortorum* (red2)	58.98	±	2.29	203.96	±	5.41	84.39	±	1.07	576.72	±	8.13
43		*Pelargonium hortorum* (red-white)	76.63	±	9.23	316.99	±	17.67	89.69	±	1.46	785.63	±	13.40
44	Lamiaceae	*Salvia microphylla* (blue)	134.76	±	3.26	293.39	±	13.02	53.73	±	0.91	452.66	±	9.08
45	Malvaceae	*Hibiscus rosa-sinensis* (orange1)	1034.10	±	37.81	348.88	±	10.47	88.02	±	2.34	755.24	±	20.97
46		*Hibiscus rosa-sinensis* (orange2)	430.61	±	7.48	257.00	±	15.57	52.78	±	7.77	441.30	±	69.59
47		*Hibiscus rosa-sinensis* (pink 1)	116.47	±	0.55	253.52	±	19.15	53.33	±	9.25	449.54	±	84.35
48		*Hibiscus rosa-sinensis* (pink 2)	137.86	±	2.64	207.38	±	15.31	74.85	±	5.49	641.90	±	48.50
49		*Hibiscus rosa-sinensis* (red 1)	125.36	±	0.77	343.84	±	16.84	75.80	±	12.67	649.25	±	2.05
50		*Hibiscus rosa-sinensis* (red 2)	314.62	±	0.76	152.00	±	8.54	83.69	±	0.29	571.38	±	2.19
51		*Hibiscus rosa-sinensis* (yellow)	2468.50	±	6.75	353.82	±	6.85	62.50	±	14.56	529.24	±	93.78
52		*Malvaviscus arboreus* (red)	88.76	±	5.86	178.51	±	15.79	78.61	±	0.97	672.06	±	9.98
53	Nyctaginaceae	*Mirabilis jalapa* (fuchsia)	277.88	±	1.67	119.36	±	0.24	85.42	±	1.06	446.95	±	85.86
54	Onagraceae	*Fuchsia magellanica* (pink)	89.30	±	1.00	174.30	±	13.09	52.21	±	2.21	441.09	±	21.35
55	Plantaginaceae	*Antirrhinum majus* (red)	230.88	±	2.37	200.53	±	6.76	84.09	±	0.59	626.48	±	5.37
56		*Antirrhinum majus* (yellow)	399.89	±	5.92	101.32	±	4.69	38.60	±	2.42	315.22	±	22.46
57	Rosaceae	*Rosa banksiae* (white)	150.51	±	1.57	199.68	±	11.96	87.25	±	5.55	753.98	±	46.32
58		*Rosa canina* (pink)	93.39	±	0.27	194.49	±	9.51	88.73	±	0.17	775.30	±	3.41
59		*Rosa damascene* (pink)	132.55	±	5.38	107.02	±	1.83	85.72	±	0.91	746.96	±	7.42
47		*Hibiscus rosa-sinensis* (pink 1)	116.47	±	0.55	253.52	±	19.15	53.33	±	9.25	449.54	±	84.35
60		*Rosa x hybrid* big pink	79.36	±	1.06	86.13	±	0.32	86.74	±	0.71	526.08	±	5.20
61		*Rosa x hybrid* big red	69.75	±	3.00	150.34	±	0.24	86.42	±	0.28	523.79	±	2.05
62		*Rosa x hybrid big white*	74.23	±	1.42	203.63	±	1.75	86.99	±	0.50	527.97	±	3.65
63		*Rosa x hybrid* big yellow	600.70	±	1.78	69.23	±	0.24	86.32	±	0.57	523.00	±	4.18
64		*Rosa x hybrid* medium (orange-yellow)	289.21	±	5.38	110.67	±	6.67	84.03	±	0.44	488.71	±	2.93
65		*Rosa x hybrid* medium orange	143.24	±	0.97	324.84	±	13.96	85.17	±	0.84	496.33	±	5.63
66		*Rosa x hybrid* medium pink	66.94	±	1.52	361.29	±	6.90	85.68	±	1.04	603.24	±	7.93
67		*Rosa x hybrid* medium purple	81.31	±	0.55	350.94	±	6.05	82.94	±	0.67	481.43	±	4.48
68		*Rosa x hybrid* medium red	52.16	±	1.64	160.88	±	6.14	85.24	±	0.32	599.93	±	2.43
69		*Rosa x hybrid* medium white	89.70	±	2.89	359.12	±	9.45	83.94	±	0.26	488.12	±	1.70
70		*Rosa x hybrid* medium yellow	206.10	±	0.39	363.90	±	8.40	83.70	±	0.53	486.51	±	3.52
71		*Rosa x hybrid* mini red	116.33	±	0.28	586.37	±	26.34	83.89	±	0.73	487.78	±	4.85
72		*Rosa x hybrid* mini orange	660.13	±	0.69	256.64	±	0.98	86.24	±	0.45	534.64	±	3.20
73		*Rosa x hybrid* mini red	55.31	±	6.84	307.49	±	0.99	85.69	±	0.85	425.19	±	94.67
74		*Rosa x hybrid* mini yellow	512.81	±	8.57	195.20	±	3.50	85.33	±	0.65	422.87	±	8.13
75		*Rosa x hybrid* roseta medium orange-yellow	209.09	±	11.52	150.09	±	2.73	82.56	±	0.60	478.90	±	3.99
76		*Rosa x hybrid* roseta medium orange	107.67	±	5.33	265.97	±	10.40	84.55	±	0.93	492.18	±	6.22
77		*Rosa x hybrid* roseta medium purple	131.93	±	0.91	246.13	±	6.19	84.39	±	0.65	491.08	±	4.36
78		*Rosa x hybrid* roseta medium pink	61.33	±	1.27	164.46	±	5.34	85.23	±	0.27	599.82	±	2.08
79		*Rosa x hybrid* roseta medium red	64.76	±	4.69	275.10	±	14.15	86.18	±	0.74	503.01	±	4.91
80		*Rosa x hybrid* roseta mini pink	107.06	±	4.35	323.12	±	7.17	84.90	±	0.79	494.47	±	5.25
81		*Rosa x hybrid* roseta mini white	87.51	±	2.86	225.17	±	6.76	84.63	±	0.40	492.69	±	2.65
82		*Rosa x hybrid* roseta big purple	56.49	±	0.55	290.73	±	1.47	82.74	±	0.65	577.14	±	5.17
83		*Rosa x hybrid* roseta big red	122.44	±	0.1	259.55	±	11.65	88.02	±	0.85	761.98	±	11.56
84		*Rosa x hybrid* rosetabig pink-white	103.33	±	0.22	223.22	±	13.80	87.43	±	1.21	761.88	±	8.52
85		*Rosa x hybrid* roseta big yellow	361.80	±	15.57	96.51	±	0.40	34.34	±	0.71	276.79	±	6.31
86	Rutaceae	*Citrus x aurantifolia* (white)	96.61	±	3.55	65.40	±	6.39	100.00	±	0.00	874.81	±	6.72
87		*Ruda chalepensis* (yellow)	923.26	±	13.68	217.59	±	6.60	90.26	±	0.79	784.56	±	8.54
88	Verbenaceae	*Aloysia citriodora* (fuchsia)	173.28	±	5.08	222.85	±	8.11	84.18	±	0.26	552.12	±	1.91
89		*Lantana camara* multicolor	2947.72	±	34.92	182.40	±	3.27	40.70	±	0.73	332.60	±	6.33
90		*Lantana viburnoides* (red)	197.57	±	0.69	259.28	±	2.57	80.36	±	1.00	545.42	±	7.86
91		*Lantana viburnoides* (red-orange)	658.02	±	3.42	224.94	±	12.33	13.14	±	5.33	82.93	±	3.51
92		*Lantana viburnoides* (yellow)	211.53	±	0.82	65.98	±	2.04	76.08	±	1.04	511.61	±	8.23
93		*Lantana viburnoides* (white)	42.81	±	2.49	101.39	±	1.67	78.51	±	0.57	459.60	±	8.95

Note: GAE, gallic acid equivalent; TE, Trolox equivalent.

**Table 4:** Average values of individual organic acids, phenolics, and carotenoids of selected flowers.

	High Concentrations of Total Carotenoids																		High Concentrations of Total Phenolics								
	(3) *C. x hybrid* (Orange)			(6) *H. annuus* (Yellow)			(7) *T. patula* (Orange)			(12) *C. indica* (Red)			(45) *H. rosa-sinensis* (Orange1)			(51) *H. rosa-sinensis* (Yellow)			(19) *P. hortorum* (Orange2)			(49) *H. rosa-sinensis* (Red1)			(70) *Rosa x hybrid* Medium Red		
Organic acid (mg/g DW)																											
Tartaric acid	23.0	±	1.6 ^f^	21.0	±	0.5 ^f^	32.4	±	0.4 ^d^	113.8	±	1.6 ^a^	18.2	±	0.4 ^g^	11.2	±	1.2 ^h^	48.8	±	1.2 ^b^	29.6	±	0.0 ^e^	37.3	±	0.0 ^c^
Malic acid	13.0	±	1.3 ^d^	24.1	±	0.6 ^c^	7.4	±	0.5 ^e^	81.7	±	3.3 ^b^	6.2	±	0.2 ^e^	9.2	±	0.1 ^e^	119.7	±	1.2 ^a^	12.4	±	0.5 ^d^	26.3	±	3.4 ^c^
Citric acid	140.5	±	0.4 ^e^	8.9	±	0.4 ^g^	11.0	±	0.1 ^fg^	11.5	±	0.3 ^fg^	910.7	±	6.8 ^b^	931.9	±	4.1 ^c^	301.5	±	7.6 ^d^	991.4	±	2.1 ^a^	18.2	±	1.7 ^f^
Total	176.5	±	0.8 ^f^	54.0	±	1.5 ^h^	50.9	±	0.2 ^h^	206.9	±	5.3 ^e^	1127.1	±	6.6 ^b^	952.3	±	2.1 ^c^	470.0	±	10.0 ^d^	1233.4	±	0.0 ^a^	18.2	±	1.7 ^g^
Phenolics (mg/100 g DW)																											
Gallic acid	2.8	±	0.0 ^c^	0.5	±	0.0 ^d^				0.1	±	0.0 ^e^	0.3	±	0.0 ^e^	0.2	±	0.0 ^e^	17.2	±	1.4 ^a^	0.6	+	0.0 ^d^	5.6	+	0.1 ^b^
Vanillic acid				0.6	±	0.0 ^a^				0.2	±	0.0 ^b^				0.5	±	0.0 ^a^									
*p*-Cumaric acid				2.8	±	0.0 ^b^													38.9	±	0.7 ^a^						
*m*-Cumaric acid				27.4	±	0.1 ^b^							52.0	±	0.8 ^a^										18.0	+	0.6 ^c^
Siringic acid													3.9	±	0.4												
Chlorogenic acid										0.8	±	0.1 ^b^				1.2	±	0.1 ^a^									
Caffeic acid				2.2	±	0.0 ^b^										3.6	±	0.5 ^a^									
Naringin										8.7	±	1.7 ^d^	46.1	±	1.5 ^b^	120.1	±	4.2 ^a^				24.9	+	1.2 ^c^			
Ferulic acid				13.0	±	0.3 ^a^				2.5	±	0.1 ^b^															
4-Hidroxy benzoic acid	476.6	±	4.7 ^b^							0.3	±	0.0 ^d^							936.2	±	32.8 ^a^	260.7	+	0.4 ^c^			
Rutin	520.1	±	19.9 ^b^							113.6	±	20.8 ^e^	465.9	±	5.7 ^c^	854.3	±	22.8 ^a^				240.5	+	8.8 ^d^			
Kampherol				45.7	±	1.9	971.9	±	8.9 ^a^	83.7	±	6.5 ^e^	801.6	±	77.2 ^b^	607.2	±	34.1 ^c^				639.7	+	30.6 ^c^	163.2	+	7.8 ^d^
Quercetin glucoside	958.8	±	9.5 ^a^	82.8	±	0.5 ^d^	936.2	±	79.2 ^a^				854.4	±	86.8 ^b^							232.6	+	12.2 ^c^	843.8	+	8.5 ^b^
Quercetin							919.3	±	43.3 ^a^	322.3	±	7.5 ^d^	459.5	±	21.9 ^c^	170.6	±	1.6 ^e^				307.2	+	15.8 ^d^	614.0	+	25.7 ^b^
Total	1958.3	±	5.8 ^c^	174.9	±	2.2 ^h^	2827.4	±	44.8 ^a^	532.2	±	18.3 ^g^	2683.7	±	17.7 ^b^	1757.7	±	9.2 ^d^	992.4	±	33.5 ^f^	1706.2	±	68.9 ^de^	1644.7	±	41.3 ^e^
Carotenoids (mg/100 g DW)																											
α-carotene	2.4	±	0.0 ^f^	604.0	±	2.1 ^b^	989.5	±	1.5 ^a^	283.6	±	1.7 ^c^	48.0	+	0.1 ^d^	34.3	+	0.3 ^e^									
β-carotene	7.2	±	0.0 ^e^	2.4	±	0.0 ^f^	601.2	±	1.9 ^a^	372.6	±	1.1 ^b^	41.6	+	0.1 ^c^	13.2	+	0.1 ^d^				2.5	+	0.0 ^f^			
β-cryptoxanthin	22.2	±	0.1 ^a^				34.2									12.1	+	0.1 ^b^									
Violaxanthin				59.5	±	0.2 ^a^	39.6	±	0.1 ^b^	23.16	±	0.1 ^c^															
Zeaxanthin				25.7	±	0.1 ^b^	149.5	±	0.0 ^a^	4.3	±	0.3 ^e^	12.0	+	0.0 ^d^	8.9	+	0.1 ^c^									
Lutein	2.8	±	0.0 ^f^	300.9	±	1.1 ^a^	219.3	±	0.1 ^b^	19.5	±	0.4 ^c^	3.7	+	0.0 ^e^	5.2	+	0.0 ^d^									
Zeinoxanthin													3.1	+	0.0 ^a^	1.9	+	0.0 ^b^									
Total	34.6	±	0.2 ^f^	1187.7	±	4.2 ^b^	703.1	±	3.7 ^c^	2043.3	±	3.6 ^a^	108.3	±	0.2 ^d^	75.6	±	0.7 ^e^	lnd			2.5	±	0.0 ^g^	lnd		

Note: The lower case letters next to the standard deviation indicate the separation of the mean values at a 95% confidence level. lnd, limit not detectable.

**Table 5:** Average values of the inhibition index of flower extracts against bacteria and fungi.

N°		Zone of Inhibition (mm)																	
		Bacterial Strain												Fungal Strain					
	Flower Extracts	*E. coli* ATCC 8739			*S. aureus* ATCC 6538P			*P. aeruginosa* ATCC 9027			*S. mutans* ATCC 25175			*C. albicans* ATCC 1031			*C. tropicalis* ATCC 13803		
3	*C. x hybrid* (orange)	-			12.5	±	0.1	11.0	±	0.0	-			14.0	±	0.0	-		
6	*H. annuus* (yellow)	-			9.5	±	0.1	-			16.0	±	0.0	-			-		
7	*T. patula* (orange)	6.0	±	0.1	15.5	±	0.0	7.0	±	0.1	20.0	±	0.0	-			-		
12	*C. indica* (red)	-			-			-			-			-			-		
45	*H. rosa-sinensis* (orange1)	7.0	±	0.1	10.5	±	0.0	12.3	±	0.1	10.0	±	0.0	-			-		
51	*H. rosa-sinensis* (yellow)	-			10.5	±	0.1	-			-			-			-		
89	*L. camara*	-			10.0	±	0.0	15.5	±	0.0	-			-			-		
19	*P. hortorum* (orange2)	11.0	±	0.2	18.0	±	0.0	15.0	±	0.0	21.0	±	0.0	8.0	±	0.0	10.0	+	0.1
49	*H. rosa-sinensis* (red1)	-			-			-			15.0	±	0.0	-			-		
70	*Rosa x hybrid* medium red	12.0	±	0.0	17.5	±	0.0	13.0	±	0.0	-			-			-		
	Control *	22.0	±	0.2	24.0	±	1.7	23.6	±	1.4	29.5	±	3.7	18.0	±	0.0	14.0	+	0.0

Note: -, non-active at the tested concentrations; *, streptomycin for bacteria and fluconazole for fungi.

## Data Availability

The original contributions presented in this study are included in the article/Supplementary Materials. Further inquiries can be directed to the corresponding author.

## Supplementary Materials

The following supporting information can be downloaded at: https://www.mdpi.com/article/10.3390/antiox13111297/s1, Table S1: Average values of the physicochemical characterisation of the flowers under study. Table S2: CIELAB colour coordinates of the flowers under study.
