# Supplementary material for: Functional, Antioxidant, Antibacterial, and Antifungal Activity of Edible Flowers
Source: Antioxidants (Basel). 2024 Oct 25;13(11):1297. doi: 10.3390/antiox13111297 (PMC11590945; doi:10.3390/antiox13111297)
Supplement: Supplementary file 1 [file antioxidants-13-01297-s001.zip › antioxidants-3246966-supplementary.pdf]

Suplementary Materials

Table S1. Average values of the physicochemical characterisation of the flowers under study.

N°	Family	Scientific name	Weight (g)	LD (cm)	ED (cm)	pH	SS (°Brix)	TA (%)	Humidity (%)	Ash (%)
1	Apiaceae	*Anethum graveolens* (yellow)	0.01± 0.00	0.20± 0.04	0.16± 0.04	6.8± 0.26	1.12± 0.10	1.02±0.08	71.00± 0.08	2.09± 0.09
2	Asteraceae	*Chrysanthemum x hybrid* (pink)	0.93± 0.15	2.96± 0.24	4.54± 0.59	12.00± 0.00	3.70± 0.48	0.35±0.06	86.03± 0.97	0.94± 0.09
3		*Chrysanthemum x hybrid* (orange)	1.90± 0.39	2.65± 0.90	4.78± 0.83	6.00± 0.00	4.70± 0.48	0.21±0.03	89.79± 0.35	0.51± 0.02
4		*Chrysanthemum x hybrid* (yellow)	3.75± 1.32	2.10± 0.23	6.12± 0.72	5.00± 0.00	4.60± 0.52	0.23±0.03	89.41± 0.15	1.05± 0.13
5		*Chrysanthemum x hybrid* (yellow-double)	3.42± 1.71	4.57± 1.19	8.72± 2.24	5.00± 0.00	3.30± 0.48	0.28±0.03	89.08± 0.03	0.68± 0.06
6		*Helianthus annuus* (yellow)	13.40± 2.71	5.23± 0.27	9.27± 0.40	6.00± 0.00	6.44± 0.26	0.09±0.00	85.92± 0.38	1.11± 0.03
7		*Tagetes patula* (orange)	6.36± 0.68	3.80± 1.58	6.52± 0.54	6.00± 0.00	4.15± 0.24	1.98±0.02	85.74± 2.14	0.81± 0.06
8		*Tagetes patula* (yellow)	3.66± 0.18	3.06± 0.46	5.88± 0.76	6.70± 0.48	2.00± 0.00	1.29±0.21	87.14± 1.85	2.27± 0.04
9	Begoniaceae	*Begonia doblet* (pink)	0.32± 0.08	0.97± 0.64	2.18± 0.52	1.88± 0.19	1.00± 0.00	1.03±0.18	97.25± 0.19	0.29± 0.00
10	Brasicaceae	*Raphanus raphanistrum* (pink)	0.29± 0.05	1.14± 0.10	1.19± 0.26	13.00± 0.00	11.00± 0.00	0.55±0.06	81.15± 0.73	3.72± 0.32
11	Cannaceae	*Canna indica* (red-double)	4.90± 2.19	3.13± 0.66	3.65± 0.80	5.50± 0.41	4.00± 0.00	0.19±0.00	89.37± 2.90	0.70± 0.08
12		*Canna indica* (red)	1.63± 0.26	7.76± 0.88	6.54± 1.78	5.00± 0.00	5.00± 0.00	0.16±0.03	97.25± 19.47	1.17± 0.02
13		*Canna indica* (yellow-orange)	1.73± 0.10	2.51± 0.46	1.91± 0.38	5.60± 0.52	3.00± 0.00	0.52±0.02	92.42± 0.45	1.19± 0.05
14	Caryophyllaceae	*Dianthus chinensis* (pink)	0.41± 0.09	3.48± 0.23	3.23± 0.59	12.40± 0.52	8.27± 1.42	0.32±0.00	85.62± 3.08	1.35± 0.05
15		*Dianthus chinensis* (red)	0.22± 0.03	2.35± 0.10	3.56± 0.06	13.00± 0.04	7.30± 0.48	4.69±0.85	95.49± 0.33	2.62± 0.15
16		*Pelargonium hortorum* (fuchsia1)	0.25± 0.07	2.08± 0.60	2.08± 1.82	2.00± 0.00	6.00± 0.52	2.67±0.08	88.78± 0.14	0.72± 0.01
17		*Pelargonium hortorum* (fuchsia2)	0.13± 0.05	3.30± 0.08	2.98± 0.12	1.26± 0.62	7.10± 1.85	0.68±0.10	87.62± 0.33	0.32± 0.06
18		*Pelargonium hortorum* (orange1)	0.22± 0.04	1.85± 0.28	1.50± 0.83	2.00± 0.00	8.60± 0.52	2.80±0.26	84.47± 0.27	0.54± 0.09
19		*Pelargonium hortorum* (orange2)	0.28± 0.01	3.66± 0.13	3.14± 0.13	3.00± 0.00	6.10± 1.85	0.74±0.07	94.73± 5.15	0.83± 0.02

Note: LD, Longitudinal diameter; ED, Ecuatorial diameter; SS, soluble solid; TA, titrable acidity

Table S1 Continue. Average values of the physicochemical characterisation of the flowers under study. Continued

N°	Family	Scientific name	Weight (g)	LD (cm)	ED (cm)	pH	SS (°Brix)	TA (%)	Humidity (%)	Ash (%)
20	Caryophyllaceae	*Pelargonium hortorum* (pink1)	0.21± 0.06	1.68± 0.34	2.60± 0.12	3.00± 0.00	9.00± 0.07	3.85±0.20	86.82± 0.15	1.63± 0.00
21		*Pelargonium hortorum* (pink2)	0.18± 0.04	1.54± 0.21	0.69± 0.10	2.00± 0.00	7.30± 0.48	1.46±0.03	84.91± 0.12	1.63± 0.25
22		*Pelargonium hortorum* (pink3)	0.24± 0.05	1.88± 0.41	2.19± 0.89	2.00± 0.03	7.60± 0.52	2.46±0.33	89.08± 0.37	0.72± 0.02
23		*Pelargonium hortorum* (pink4)	0.12± 0.04	3.14± 0.07	2.96± 0.18	2.00± 0.01	4.50± 1.35	0.37±0.04	95.10± 3.12	0.41± 0.05
24		*Pelargonium hortorum* (pink-fuchsia)	0.25± 0.03	2.53± 0.26	3.24± 0.91	3.00± 0.02	8.00± 0.52	1.48±0.19	87.45± 0.22	2.03± 0.07
25		*Pelargonium hortorum* (pink-white1)	0.31± 0.02	1.77± 0.58	3.38± 1.40	1.00± 0.00	8.00± 0.00	1.47±0.09	88.17± 0.90	1.00± 0.01
26		*Pelargonium hortorum* (pink-white2)	0.26± 0.02	2.32± 0.26	2.64± 1.49	0.80± 0.26	8.45± 0.44	1.50±0.19	87.61± 0.72	1.44± 0.06
27		*Pelargonium hortorum* (red1)	0.15± 0.03	1.43± 0.36	0.85± 0.55	2.00± 0.00	7.00± 0.48	3.02±0.21	86.70± 0.54	1.47± 0.07
28		*Pelargonium hortorum* (red2)	0.35± 0.10	2.70± 0.55	3.91± 1.72	2.00± 0.52	9.00± 0.31	1.14±0.08	86.20± 0.18	1.12± 0.02
29		*Pelargonium hortorum* (white1)	0.22± 0.05	2.01± 0.42	1.44± 0.31	2.00± 0.00	9.00± 0.76	1.03±0.03	83.81± 0.98	1.21± 0.08
30		*Pelargonium hortorum* (white2)	0.12± 0.04	2.38± 0.28	2.45± 0.22	5.18± 0.29	8.20± 1.03	1.13±0.04	90.24± 0.44	0.14± 0.07
31	Compositae	*Calendula officinalis*(yellow)	2.03± 0.43	2.52± 0.31	2.53± 0.18	4.45± 0.44	2.15± 0.18	1.51±0.39	85.52± 1.37	0.66± 0.03
32		*Chamaemelun nobile* (white)	0.11± 0.00	1.19± 0.18	1.86± 0.27	5.80± 0.42	2.66± 0.85	0.31±0.02	91.69± 0.45	0.54± 0.09
33		*Dahlia pinnata* (fuchsia1)	11.4± 0.48	2.11± 1.75	7.63± 0.10	4.15± 0.24	3.92± 0.10	0.22±0.07	85.28± 4.14	0.75± 0.00
34		*Dahlia pinnata* (fuchsia2)	7.89± 0.48	3.12± 0.12	5.99± 0.75	5.45± 0.37	2.37± 0.46	0.23±0.02	84.03± 0.21	0.27± 0.01
35		*Dahlia pinnata* (orange)	8.39± 0.68	3.17± 0.31	15.57± 2.86	4.45± 0.44	2.74± 0.10	0.12±0.01	90.50± 0.06	0.62± 0.01
36		*Dahlia pinnata* (red)	12.60± 1.43	2.98± 0.20	11.97± 0.70	6.32± 0.23	2.00± 0.26	0.16±0.03	91.14± 0.37	1.50± 0.04
37	Fabaceae	*Trifolium repens* (white)	0.22± 0.01	1.59± 0.82	1.90± 0.44	6.50± 0.53	2.35± 0.05	0.31±0.02	75.42± 4.23	1.97± 0.29
38	Geraniaceae	*Pelargonium hortorum* (fuchsia1)	0.36± 0.10	2.03± 0.23	3.83± 0.59	4.05± 0.93	2.30± 0.48	0.55±0.07	88.44± 0.17	1.05± 0.11
39		*Pelargonium hortorum* (fuchsia2)	0.46± 0.01	1.79± 0.26	4.76± 0.08	4.45± 0.44	6.65± 0.24	1.31±0.24	84.14± 0.71	1.52± 0.10
40		*Pelargonium hortorum* (pink)	0.07± 0.01	1.60± 0.21	1.49± 0.19	3.30± 0.48	0.20± 0.00	0.46±0.06	95.16± 0.27	0.62± 0.09
41		*Pelargonium hortorum* (red1)	0.23± 0.01	1.08± 0.09	5.25± 0.22	2.00± 0.23	4.00± 0.00	0.71±0.03	88.59± 0.11	1.08± 0.03
42		*Pelargonium hortorum* (red2)	0.16± 0.01	2.90± 0.17	3.71± 0.30	13.00± 0.00	4.00± 0.00	0.83±0.28	73.33± 0.28	0.72± 0.18
43		*Pelargonium hortorum* (red-white)	0.07± 0.00	3.06± 0.42	1.86± 0.07	3.70± 0.48	1.00± 0.00	3.21±0.80	51.87± 1.23	1.35± 0.08
44	Lamiaceae	*Salvia microphylla* (blue)	0.02± 0.00	1.01± 0.39	0.29± 0.01	6.00± 0.19	1.00± 0.00	0.38±0.00	84.98± 3.73	0.44± 0.04
45	Malvaceae	*Hibiscus rosa-sinensis* (orange1)	4.53± 0.74	7.68± 0.14	9.17± 1.95	5.70± 0.48	5.00± 0.00	0.42±0.02	88.47± 7.27	1.40± 0.04
46		*Hibiscus rosa-sinensis* (orange2)	4.63± 0.70	7.80± 0.30	8.77± 2.15	6.00± 0.00	5.97± 0.05	0.96±0.03	81.72± 0.75	1.96± 0.04
47		*Hibiscus rosa-sinensis* (pink 1)	2.83± 0.31	12.15± 0.77	7.27± 1.23	5.00± 0.00	3.07± 0.05	0.87±0.04	81.51± 31.22	1.79± 0.14
48		*Hibiscus rosa-sinensis* (pink 2)	2.80± 0.47	8.88± 1.45	5.71± 0.89	6.00± 0.26	1.09± 0.10	0.80±0.08	85.34± 0.02	0.67± 0.09
49		*Hibiscus rosa-sinensis* (red 1)	6.73± 0.10	11.01± 0.51	9.08± 0.69	8.00± 0.00	1.16± 0.11	0.90±0.17	81.13± 0.28	2.74± 0.14
50		*Hibiscus rosa-sinensis* (red 2)	2.01± 0.16	7.48± 0.67	6.22± 0.79	1.00± 0.52	2.60± 0.52	0.21±0.08	87.18± 0.24	0.79± 0.08
51		*Hibiscus rosa-sinensis* (yellow)	5.03± 0.93	7.51± 0.54	9.20± 2.12	5.00± 0.29	5.32± 0.23	0.16±0.02	80.19± 0.86	2.67± 0.17
52		*Malvaviscus arboreus* (red)	1.65± 0.26	8.11± 0.52	1.47± 0.23	5.50± 0.41	2.30± 0.48	1.57±0.10	91.57± 0.34	0.21± 0.01

Note: LD, Longitudinal diameter; ED, Ecuatorial diameter; SS, soluble solid; TA, titrable acidity

Table S1 Continue. Average values of the physicochemical characterisation of the flowers under study. Continued

N°	Family	Scientific name	Weight (g)	LD (cm)	ED (cm)	pH	SS (°Brix)	TA (%)	Humidity (%)	Ash (%)
53	Nyctaginaceae	*Mirabilis jalapa* (fuchsia)	0.10± 0.00	2.86± 0.27	0.49± 0.07	5.50± 0.00	5.30± 0.48	0.25±0.00	80.02± 1.38	1.38± 0.11
54	Onagraceae	*Fuchsia magellanica* (pink)	0.73± 0.01	6.20± 0.41	3.05± 0.74	3.60± 0.52	2.63± 0.31	0.43±0.08	92.58± 0.58	1.42± 0.08
55	Plantaginaceae	*Antirrhinum majus* (red)	0.29± 0.03	3.94± 0.49	2.68± 0.73	13.00± 0.00	5.60± 0.52	2.59±0.20	85.49± 2.55	2.75± 0.19
56		*Antirrhinum majus* (yellow)	0.60± 0.09	4.53± 0.29	2.70± 0.63	5.60± 0.97	8.36± 1.83	0.06±0.00	83.66± 1.60	6.79± 0.59
57	Rosaceae	*Rosa banksiae* (white)	3.01± 0.87	5.08± 1.29	6.61± 1.90	5.00± 0.00	4.55± 0.44	2.05±0.00	81.42± 0.27	1.35± 0.01
58		*Rosa canina* (pink)	0.71± 0.05	2.56± 0.03	2.29± 0.26	3.45± 0.44	4.97± 0.05	0.15±0.02	82.30± 0.58	0.36± 0.02
59		*Rosa damascene* (pink)	1.12± 0.21	5.92± 0.40	1.50± 0.33	5.00± 0.37	5.50± 0.41	1.08±0.07	81.51± 0.26	0.66± 0.03
60		*Rosa x hybrid* big pink	22.79± 4.54	4.55± 0.36	2.78± 0.30	3.00± 0.00	6.00± 0.00	0.28±0.04	79.36± 1.06	1.17± 0.16
61		*Rosa x hybrid* big red	23.39± 5.25	4.13± 0.51	2.47± 0.40	3.00± 0.26	6.30± 0.48	0.26±0.02	84.68± 0.25	0.10± 0.01
62		*Rosa x hybrid big white*	22.79± 4.54	4.56± 0.28	2.54± 0.28	5.30± 0.48	6.00± 0.05	0.30±0.01	87.33± 0.20	1.00± 0.09
63		*Rosa x hybrid* big yellow	18.71± 1.05	3.79± 0.16	2.30± 0.15	6.00± 0.00	5.00± 0.52	0.28±0.04	83.49± 1.99	1.90± 0.14
64		*Rosa x hybrid* medium (orange-yellow)	2.92± 0.12	1.75± 0.54	5.59± 0.94	4.60± 0.52	4.04± 0.81	0.85±0.01	80.31± 3.70	2.00± 0.29
65		*Rosa x hybrid* medium orange	1.89± 0.05	1.09± 0.16	4.52± 0.25	4.00± 0.00	8.32± 0.13	2.60±0.20	83.72± 0.20	0.67± 0.08
66		*Rosa x hybrid* medium pink	1.16± 0.14	1.34± 0.25	5.18± 0.06	2.00± 0.00	7.00± 0.85	3.83±0.33	84.04± 1.44	1.43± 0.01
67		*Rosa x hybrid* medium purple	3.09± 0.01	2.86± 0.26	5.75± 0.16	5.00± 0.48	3.22± 0.10	3.84±0.11	86.04± 2.14	0.34± 0.09
68		*Rosa x hybrid* medium red	1.57± 0.24	2.25± 0.48	4.67± 1.43	3.00± 0.00	4.99± 0.09	1.64±0.09	83.70± 2.11	1.25± 0.11
69		*Rosa x hybrid* medium white	2.99± 0.42	1.89± 0.04	6.79± 0.18	6.00± 0.00	3.00± 0.00	0.92±0.01	84.19± 2.74	7.23± 0.07
70		*Rosa x hybrid* medium yellow	4.75± 0.23	1.41± 0.00	7.31± 0.46	4.45± 0.44	5.17± 0.05	1.49±0.22	85.81± 1.92	1.17± 0.01
71		*Rosa x hybrid* mini red	1.38± 0.04	0.82± 0.05	3.83± 0.08	2.60± 0.52	2.06± 0.05	0.94±0.04	83.34± 0.36	1.29± 0.03
72		*Rosa x hybrid* mini orange	3.40± 0.95	3.03± 0.05	2.97± 0.11	3.80± 0.03	7.48± 0.21	0.27±0.06	84.18± 0.49	0.96± 0.07
73		*Rosa x hybrid* mini red	3.77± 0.27	3.29± 0.15	2.91± 0.66	1.00± 0.01	7.16± 0.05	0.53±0.08	86.10± 0.26	0.57± 0.07
74		*Rosa x hybrid* mini yellow	3.17± 0.37	2.75± 0.09	2.90± 0.16	5.00± 0.10	6.00± 0.88	0.56±0.04	86.38± 0.22	0.92± 0.03
75		*Rosa x hybrid* roseta medium orange-yellow	2.12± 0.12	1.60± 0.31	4.94± 0.14	4.60± 0.52	3.00± 0.05	0.25±0.04	80.29± 0.40	1.10± 0.07
76		*Rosa x hybrid* roseta medium orange	5.09± 0.10	1.89± 0.02	5.87± 0.27	3.00± 0.04	8.00± 0.24	2.52±0.05	82.58± 1.12	0.85± 0.08
77		*Rosa x hybrid* roseta medium purple	1.01± 0.09	0.99± 0.01	4.13± 0.10	4.00± 0.00	3.50± 0.05	2.80±0.36	83.65± 0.33	0.66± 0.09
78		*Rosa x hybrid* roseta medium pink	3.03± 0.04	3.45± 0.18	6.36± 0.19	10.30± 0.48	4.30± 0.48	0.45±0.13	87.36± 1.07	0.66± 0.02
79		*Rosa x hybrid* roseta medium red	2.03± 0.28	1.30± 0.09	4.74± 0.16	2.00± 0.48	10.00± 0.30	1.92±0.18	81.42± 0.16	1.67± 0.03
80		*Rosa x hybrid* roseta mini pink	2.29± 0.27	1.29± 0.15	5.59± 0.66	2.30± 0.48	6.87± 0.05	2.06±0.28	82.12± 0.02	3.37± 0.17
81		*Rosa x hybrid* roseta mini white	1.47± 0.27	0.96± 0.05	3.36± 0.11	5.00± 0.00	3.84± 0.21	0.95±0.17	79.33± 0.15	1.36± 0.07
82		*Rosa x hybrid* roseta big purple	12.30± 0.04	8.07± 0.04	5.38± 0.03	3.00± 0.00	3.17± 0.05	0.61±0.02	82.68± 0.03	0.94± 0.08
83		*Rosa x hybrid* roseta big red	10.75± 0.35	4.90± 0.74	9.20± 0.32	10.80± 0.26	11.00± 0.44	0.27±0.08	86.68± 0.59	0.93± 0.05
84		*Rosa x hybrid* rosetabig pink-white	10.05± 1.35	6.98± 0.05	6.82± 1.06	2.75± 0.26	5.97± 0.05	0.21±0.03	83.89± 0.70	0.71± 0.01
85		*Rosa x hybrid* roseta big yellow	6.85± 0.18	4.57± 0.58	8.85± 0.50	4.75± 0.26	4.00± 0.00	0.30±0.07	83.22± 0.15	0.59± 0.01
86	Rutaceae	*Citrus x aurantifolia* (white)	0.26± 0.01	1.25± 0.06	0.63± 0.076	7.70± 0.48	4.54± 0.12	0.66±0.01	83.68± 1.03	0.89± 0.05
87		*Ruda chalepensis* (yellow)	0.06± 0.01	0.92± 0.06	0.97± 0.02	6.00± 0.00	3.40± 0.52	3.49±0.36	83.66± 2.46	6.98± 0.62

88	Verbenaceae	*Aloysia citriodora* (fuchsia)	0.03± 0.00	2.71± 0.35	0.62± 0.01	6.00± 0.01	9.00± 0.02	2.19±0.05	76.62± 1.21	4.91± 0.04
89		*Lantana camara* multicolour	0.01± 0.00	1.31± 0.17	0.58± 0.08	6.00± 0.00	0.10± 0.00	4.52±0.03	86.88± 0.79	2.23± 0.20
90		*Lantana viburnoides* (red)	0.01± 0.00	1.24± 0.13	0.56± 0.13	5.90± 0.52	3.78± 0.60	0.13±0.01	85.47± 0.33	0.85± 0.12
91		*Lantana viburnoides* (red-orange)	0.01± 0.00	1.26± 0.15	0.64± 0.12	5.63± 0.46	1.41± 0.07	0.44±0.06	80.99± 0.21	2.36± 0.25
92		*Lantana viburnoides* (yellow)	0.01± 0.00	1.08± 0.12	0.71± 0.10	5.50± 0.00	4.30± 0.48	0.13±0.01	84.13± 0.31	0.09± 0.00
93		*Lantana viburnoides* (white)	0.02± 0.00	1.36± 0.09	0.64± 0.14	5.20± 0.42	4.40± 0.52	0.43±0.03	85.79± 0.16	0.78± 0.07

Note: LD, Longitudinal diameter; ED, Ecuatorial diameter; SS, soluble solid; TA, titrable acid

Table S2. CIELAB colour coordinates of the flowers under study.

N°	Family	Species	L	a*	b*
1	Apiaceae	*Anethum graveolens* (yellow)	53.1 ± 3.3	-6.9 ± 1.8	28.4 ± 3.4
2	Asteraceae	*Chrysanthemum x hybrid* (pink)	58.2 ± 1.6	30.2 ± 1.3	-9.8 ± 0.3
3		*Chrysanthemum x hybrid* (orange)	73.4 ± 0.7	5.6 ± 0.7	72.6 ± 1.9
4		*Chrysanthemum x hybrid* (yellow)	81.6 ± 1.7	-4.8 ± 0.6	75.3 ± 3.1
5		*Chrysanthemum x hybrid* (yellow-double)	79.5 ± 3.7	-6.4 ± 0.5	52.8 ± 3.6
6		*Helianthus annuus* (yellow)	75.0 ± 0.8	10.4 ± 0.8	79.2 ± 3.4
7		*Tagetes patula* (orange)	76.8 ± 1.6	1.9 ± 0.1	90.2 ± 1.8
8		*Tagetes patula* (yellow)	75.9 ± 3.9	-10.8 ± 0.9	60.1 ± 7.1
9	Begoniaceae	*Begonia doblet* (pink)	54.6 ± 1.8	22.9 ± 4.2	1.1 ± 0.1
10	Brassicaceae	*Raphanus raphanistrum* (pink)	76.6 ± 5.6	21.9 ± 9.4	-4.8 ± 0.6
11	Cannaceae	*Canna indica* (red-double)	39.1 ± 1.6	51.7 ± 3.8	39.9 ± 6.8
12		*Canna indica* (red)	42.7 ± 0.8	38.1 ± 1.1	30.3 ± 1.3
13		*Canna indica* (yellow-orange)	65.2 ± 6.1	9.3 ± 0.8	56.5 ± 6.3
14	Caryophyllaceae	*Dianthus chinensis* (pink)	54.5 ± 5.5	33.8 ± 4.3	-4.7 ± 0.5
15		*Dianthus chinensis* (red)	27.3 ± 0.8	36.1 ± 3.4	6.2 ± 1.3
16		*Pelargonium hortorum* (fuchsia1)	37.1 ± 1.1	59.1 ± 2.3	4.3 ± 0.3
17		*Pelargonium hortorum* (fuchsia2)	50.8 ± 1.3	57.5 ± 9.0	-7.9 ± 1.3
18		*Pelargonium hortorum* (orange1)	53.4 ± 2.7	56.0 ± 1.2	44.3 ± 1.9
19		*Pelargonium hortorum* (orange2)	49.5 ± 1.0	55.4 ± 2.5	50.7 ± 1.3
20		*Pelargonium hortorum* (pink1)	78.4 ± 3.0	19.8 ± 6.8	-1.5 ± 1.0
21		*Pelargonium hortorum* (pink2)	65.1 ± 2.4	43.6 ± 4.0	-8.6 ± 1.7
22		*Pelargonium hortorum* (pink3)	66.5 ± 0.8	41.0 ± 3.9	13.2 ± 3.3
23		*Pelargonium hortorum* (pink4)	65.9 ± 6.7	43.6 ± 7.1	21.0 ± 2.3
24		*Pelargonium hortorum* (pink-fuchsia)	57.8 ± 10.0	42.5 ± 10.7	6.1 ± 0.5
25		*Pelargonium hortorum* (pink-white1)	66.2 ± 9.6	33.7 ± 13.9	-15.4 ± 3.2
26		*Pelargonium hortorum* (pink-white2)	66.4 ± 3.9	42.1 ± 6.6	-2.2 ± 1.1
27		*Pelargonium hortorum* (red1)	40.4 ± 1.0	58.4 ± 2.0	27.8 ± 1.2
28		*Pelargonium hortorum* (red2)	30.2 ± 0.7	50.3 ± 1.0	15.3 ± 1.0
29		*Pelargonium hortorum* (white1)	89.8 ± 0.3	-0.4 ± 0.0	4.0 ± 0.9
30		*Pelargonium hortorum* (white2)	88.4 ± 0.8	-0.8 ± 0.4	2.5 ± 0.6
31	Compositae	*Calendula officinalis*(yellow)	59.3 ± 4.1	28.2 ± 0.8	51.1 ± 7.3
32		*Chamaemelun nobile* (white)	78.8 ± 6.6	-2.5 ± 0.1	5.9 ± 0.5
33		*Dahlia pinnata* (fuchsia1)	47.8 ± 3.5	32.6 ± 2.0	-13.8 ± 1.1
34		*Dahlia pinnata* (fuchsia2)	25.8 ± 0.3	60.9 ± 9.8	-21.7 ± 1.1
35		*Dahlia pinnata* (orange)	69.6 ± 2.4	0.9 ± 0.0	56.7 ± 2.4
36		*Dahlia pinnata* (red)	27.5 ± 1.0	50.8 ± 0.9	21.9 ± 0.8
37	Fabaceae	*Trifolium repens* (white)	69.2 ± 2.2	-4.2 ± 1.6	15.7 ± 1.2
38	Geraniaceae	*Pelargonium hortorum* (fuchsia1)	43.3 ± 2.0	52.3 ± 4.5	-9.3 ± 1.5
39		*Pelargonium hortorum* (fuchsia2)	22.2 ± 1.1	8.9 ± 4.1	-2.3 ± 0.4
40		*Pelargonium hortorum* (pink)	75.8 ± 2.7	14.6 ± 4.0	6.4 ± 1.3
41		*Pelargonium hortorum* (red1)	28.3 ± 0.5	55.8 ± 1.4	24.7 ± 0.4
42		*Pelargonium hortorum* (red2)	23.8 ± 2.5	5.6 ± 0.3	-4.5 ± 0.3
43		*Pelargonium hortorum* (red-white)	41.4 ± 2.9	42.1 ± 1.6	5.4 ± 1.5
44	Lamiaceae	*Salvia microphylla* (blue)	37.2 ± 4.9	10.2 ± 3.5	-11.3 ± 1.6
45	Malvaceae	*Hibiscus rosa-sinensis* (orange1)	49.6 ± 2.1	38.7 ± 3.4	30.3 ± 1.9
46		*Hibiscus rosa-sinensis* (orange2)	71.5 ± 2.2	13.9 ± 2.7	26.6 ± 4.3
47		*Hibiscus rosa-sinensis* (pink 1)	69.7 ± 1.5	17.7 ± 3.1	3.4 ± 2.1
48		*Hibiscus rosa-sinensis* (pink 2)	51.5 ± 2.1	31.0 ± 2.1	7.0 ± 0.4
49		*Hibiscus rosa-sinensis* (red 1)	35.9 ± 2.3	30.2 ± 1.9	5.3 ± 1.4
50		*Hibiscus rosa-sinensis* (red 2)	35.4 ± 3.3	51.6 ± 6.2	18.0 ± 5.3
51		*Hibiscus rosa-sinensis* (yellow)	74.7 ± 3.7	0.5 ± 0.0	65.7 ± 1.5

Table S2 Continue. CIELAB colour coordinates of the flowers under study. Continuation

N°	Family	Species	L	a*	b*
52		*Malvaviscus arboreus* (red)	40.7 ± 3.0	31.7 ± 0.8	18.1 ± 0.6
53	Nyctaginaceae	*Mirabilis jalapa* (fuchsia)	24.1 ± 4.3	29.3 ± 4.6	3.9 ± 1.4
54	Onagraceae	*Fuchsia magellanica* (pink)	47.2 ± 1.6	43.9 ± 3.7	28.0 ± 1.2
55	Plantaginaceae	*Antirrhinum majus* (red)	27.8 ± 0.6	22.9 ± 4.7	11.2 ± 0.4
56		*Antirrhinum majus* (yellow)	71.0 ± 2.9	-11.2 ± 1.8	81.3 ± 2.9
57	Rosaceae	*Rosa banksiae* (white)	78.1 ± 1.1	-2.2 ± 0.9	12.1 ± 1.2
58		*Rosa canina* (pink)	72.5 ± 1.8	14.1 ± 1.6	2.9 ± 0.4
59		*Rosa damascene* (pink)	81.7 ± 2.1	2.1 ± 0.9	7.9 ± 2.6
60		*Rosa x hybrid* big pink	44.8 ± 1.1	50.2 ± 0.9	-2.7 ± 1.1
61		*Rosa x hybrid* big red	29.4 ± 0.6	43.5 ± 0.8	12.5 ± 0.9
62		*Rosa x hybrid big white*	*80.6 ± 1.3*	*-1.7 ± 0.4*	*5.6 ± 0.4*
63		*Rosa x hybrid* big yellow	75.1 ± 0.5	-2.9 ± 0.7	72.5 ± 0.5
64		*Rosa x hybrid* medium (orange-yellow)	70.1 ± 2.9	13.6 ± 0.7	44.2 ± 2.6
65		*Rosa x hybrid* medium orange	54.5 ± 1.4	40.0 ± 7.9	32.6 ± 2.9
66		*Rosa x hybrid* medium pink	62.6 ± 0.5	42.4 ± 1.5	23.5 ± 1.6
67		*Rosa x hybrid* medium purple	63.6 ± 2.2	42.1 ± 5.3	1.7 ± 0.6
68		*Rosa x hybrid* medium red	39.7 ± 1.0	54.2 ± 0.7	28.6 ± 1.4
69		*Rosa x hybrid* medium white	85.5 ± 1.9	-1.7 ± 0.5	11.5 ± 1.8
70		*Rosa x hybrid* medium yellow	77.8 ± 2.8	-4.7 ± 0.9	66.8 ± 2.9
71		*Rosa x hybrid* mini red	33.8 ± 1.6	51.6 ± 6.2	20.7 ± 3.5
72		*Rosa x hybrid* mini orange	55.7 ± 0.7	31.1 ± 1.7	46.0 ± 0.8
73		*Rosa x hybrid* mini red	34.9 ± 0.9	54.4 ± 0.5	34.3 ± 2.6
74		*Rosa x hybrid* mini yellow	75.4 ± 0.3	-5.8 ± 1.0	58.8 ± 5.9
75		*Rosa x hybrid* roseta medium orange-yellow	75.5 ± 6.0	13.3 ± 0.9	43.8 ± 6.0
76		*Rosa x hybrid* roseta medium orange	52.4 ± 2.0	53.6 ± 2.4	46.5 ± 2.4
77		*Rosa x hybrid* roseta medium purple	80.5 ± 3.2	1.0 ± 0.2	8.8 ± 2.6
78		*Rosa x hybrid* roseta medium pink	45.9 ± 0.5	52.0 ± 3.0	6.2 ± 1.3
79		*Rosa x hybrid* roseta medium red	28.4 ± 1.5	50.2 ± 4.2	14.9 ± 2.5
80		*Rosa x hybrid* roseta mini pink	40.5 ± 1.0	55.5 ± 1.0	10.3 ± 1.4
81		*Rosa x hybrid* roseta mini white	76.5 ± 1.7	14.6 ± 5.5	38.5 ± 3.0
82		*Rosa x hybrid* roseta big purple	54.9 ± 0.9	40.9 ± 0.7	-4.3 ± 1.4
83		*Rosa x hybrid* roseta big red	36.6 ± 1.0	50.1 ± 1.0	20.3 ± 1.1
84		*Rosa x hybrid* rosetabig pink-white	47.6 ± 2.0	42.7 ± 2.3	3.0 ± 0.2
85		*Rosa x hybrid* roseta big yellow	74.1 ± 1.0	-6.7 ± 0.5	35.0 ± 3.2
86	Rutaceae	*Citrus x aurantifolia* (white)	82.4 ± 1.3	-2.5 ± 0.4	9.6 ± 2.9
87		*Ruda chalepensis* (yellow)	48.5 ± 3.1	-4.5 ± 1.5	36.2 ± 3.3
88	Verbenaceae	*Aloysia citriodora* (fuchsia)	78.8 ± 6.6	-2.5 ± 0.1	5.9 ± 0.5
89		*Lantana camara* multicolour	46.3 ± 1.8	24.4 ± 1.3	21.9 ± 2.3
90		*Lantana viburnoides* (red)	34.3 ± 2.1	35.7 ± 7.3	13.0 ± 3.8
91		*Lantana viburnoides* (red-orange)	55.5 ± 2.9	23.1 ± 3.0	43.7 ± 12.6
92		*Lantana viburnoides* (yellow)	76.0 ± 0.9	4.4 ± 1.6	79.6 ± 2.9
93		*Lantana viburnoides* (white)	77.9 ± 3.4	-2.2 ± 0.7	26.3 ± 3.6
